# Development of a Novel Nanoarchitecture of the Robust Photosystem I from a Volcanic Microalga *Cyanidioschyzon merolae* on Single Layer Graphene for Improved Photocurrent Generation

**DOI:** 10.3390/ijms22168396

**Published:** 2021-08-05

**Authors:** Miriam Izzo, Margot Jacquet, Takayuki Fujiwara, Ersan Harputlu, Radosław Mazur, Piotr Wróbel, Tomasz Góral, C. Gokhan Unlu, Kasim Ocakoglu, Shinya Miyagishima, Joanna Kargul

**Affiliations:** 1 Solar Fuels Laboratory, Center of New Technologies, University of Warsaw, Banacha 2C, 02-097 Warsaw, Poland; m.izzo@cent.uw.edu.pl (M.I.); m.jacquet@cent.uw.edu.pl (M.J.); 2 Department of Gene Function and Phenomics, National Institute of Genetics, Yata 111, Mishima 411-8540, Japan; tkfujiwara@nig.ac.jp (T.F.); smiyagis@nig.ac.jp (S.M.); 3 Department of Engineering Fundamental Sciences, Faculty of Engineering, Tarsus University, Tarsus 33400, Turkey; ersan.harputlu@gmail.com (E.H.); kasim.ocakoglu@tarsus.edu.tr (K.O.); 4 Department of Metabolic Regulation, Faculty of Biology, Institute of Biochemistry, University of Warsaw, Miecznikowa 1, 02-096 Warsaw, Poland; rmazur@biol.uw.edu.pl; 5 Faculty of Physics, University of Warsaw, Pasteura 5, 02-093 Warsaw, Poland; Piotr.Wrobel@fuw.edu.pl; 6 Cryomicroscopy and Electron Diffraction Core Facility, Center of New Technologies, University of Warsaw, 02-097 Warsaw, Poland; t.goral@cent.uw.edu.pl; 7 Department of Biomedical Engineering, Pamukkale University, Denizli 20070, Turkey; cunlu@pau.edu.tr

**Keywords:** biohybrid nanodevices, biophotovoltaics, *Cyanidioschyzon merolae*, direct electron transfer, photosystem I, single layer graphene

## Abstract

Here, we report the development of a novel photoactive biomolecular nanoarchitecture based on the genetically engineered extremophilic photosystem I (PSI) biophotocatalyst interfaced with a single layer graphene via pyrene-nitrilotriacetic acid self-assembled monolayer (SAM). For the oriented and stable immobilization of the PSI biophotocatalyst, an His_6_-tag was genetically engineered at the *N*-terminus of the stromal PsaD subunit of PSI, allowing for the preferential binding of this photoactive complex with its reducing side towards the graphene monolayer. This approach yielded a novel robust and ordered nanoarchitecture designed to generate an efficient direct electron transfer pathway between graphene, the metal redox center in the organic SAM and the photo-oxidized PSI biocatalyst. The nanosystem yielded an overall current output of 16.5 µA·cm^−2^ for the nickel- and 17.3 µA·cm^−2^ for the cobalt-based nanoassemblies, and was stable for at least 1 h of continuous standard illumination. The novel green nanosystem described in this work carries the high potential for future applications due to its robustness, highly ordered and simple architecture characterized by the high biophotocatalyst loading as well as simplicity of manufacturing.

## 1. Introduction

Photosynthesis is one of the most fundamental processes carried out by phototrophic organisms to convert photons into chemical energy [1]. This process evolved on earth over 3.5 billion years ago [2] and is directly responsible not only for the production of atmospheric oxygen, allowing the biosphere as we know to prosper, but it has also provided all the fossil fuels that drive the present-day economies. However, the continuous use of fossil fuels for the last 2 centuries [3] has brought about an onset of destructive climate changes due to increased CO_2_ emissions. To ameliorate climate change and to meet the ever-growing energy demand of humankind, the race is on to provide alternative viable technologies to produce sustainable fuels in a circular economy model. Of all possible forms of renewable energy available on earth, sunlight is by far the most abundant [4]. Hence, the biomimicry of the natural photosynthesis process offers an attractive technological option for solar-to-chemical conversion.

One of the most promising artificial photosynthesis approaches is based on rational nanoengineering of the biomolecular solar converting systems which encompass the robust biophotocatalysts, such as photosystem I (PSI) which works in tandem with the solar-driven water-splitting enzyme, photosystem II (PSII) to carry out the electron and proton transfer in conjunction with cytochrome *b_6_f* (cyt *b_6_f*) and the specific mobile electron carriers [5,6]. The artificial photosynthesis approach uses the natural process as a blueprint, extrapolating the fundamental principles of the early energy conversion steps and conjugating either the synthetic photocatalysts, natural (photo)enzymes or the hybrid nanostructures of both components with smart electrode materials [7,8]. In the biomolecular approach, a biophotocatalyst of choice is often the PSI complex, a highly stable macromolecular machine able to efficiently convert photons into a long-lived charge separated state working with an internal quantum efficiency close to 100% [9,10]. The PSII complex has also been applied for the same purpose in the proof-of-principle solar conversion devices reconstructing the photosynthetic *Z*-scheme, although the stability of such systems if often very limited [11,12,13]. The photons absorbed by the light-harvesting antenna subunits of the PSI complex reach the special pair of chlorophyll *a* (Chl*a*) molecules, the P700 reaction center, where the primary electron donor (P700*; E_m_ = −1.3 V vs. SHE or A_0_; E_m_ = −1.05 V vs. SHE) is generated on a ps timescale concomitant with the formation of a charge separated state and the initiation of electron transfer through a branched sequence of redox cofactors embedded in the protein scaffold [14]. Namely, from the P700* the electrons are transferred via two pairs of Chls and a pair of phylloquinones to three consecutive iron–sulfur clusters, F_X_, F_A_ and F_B_ (for the terminal F_B_ cluster E_m_ = −0.58 V vs. SHE). The oxidized reaction center P700^+^ (E_m_ = 0.43 V vs. SHE) is then re-reduced by a mobile electron carrier such as cytochrome *c*_6_ (cyt *c*_6_) [15,16,17].

The main bottleneck of the present-day artificial photosynthesis technologies is the low power conversion efficiency caused by suboptimal direct electron transfer (DET) hampered by charge recombination and short-circuiting occurring in the working modules and interfaces. A key factor to optimize and boost the overall photocurrent density output is the efficient, i.e., uniform and dense immobilization of the biophotocatalysts, such as PSI, on the electrode surface to minimize the DET losses. To this end, various approaches have been employed [18], including the manipulation of surface charge distribution of the PSI complex and electrode surface to promote the desired orientation of the biophotocatalyst [19,20], functionalization of the surface with various types of self-assembled monolayer (SAM) with different functional head groups for strong anchoring of the photosynthetic reaction center [21], integration of oriented PSI in a lipid biolayer [22], embedding PSI and PSII biophotocatalysts in redox-active hydrogels [23], domain-specific molecular recognition between the cyt *c*_6_ (natural electron donor of PSI) and the oxidizing side of PSI [24], orientation of PSI by electrodeposition [25,26] or molecular wiring of the redox cofactors of PSI (e.g., phylloquinone) with functionalized electrode surface [27]. Another powerful approach is based on genetic engineering of PSI so as to introduce the specific amino acid residues, affinity tags or peptide linkers into the structure of this biophotocatalyst for its stable interaction with the appropriately functionalized electrode surface [28,29,30].

In this work, we produced a novel biohybrid nanodevice based on single layer graphene (SLG) transferred onto a fluorine-doped tin oxide (FTO) substrate. Graphene is a monoatomic layer of carbon atoms, organized in a crystalline structure with hexagonal cells. This structure has a planar conformation making the graphene a bidimensional material. Due to the unique proprieties, such as being the only known semiconductor with a zero energy gap, the graphene has attracted a lot of interest for the research of novel materials. Among the most important physical properties that are important for application of graphene in photoelectrodes several are worth mentioning, including its transparency, elasticity, mechanical robustness, the presence of a strong ambipolar electric field effect and high electron mobility within a flat large sp^2^-hybridized carbon lattice bonding with the π-electron clouds, and electrical conductivity more than 10 times higher than copper [31,32,33,34]. These properties make graphene a perfect material of choice for the construction of efficient photoelectrodes. The simplicity of the functionalization process via the π–π stacking method makes graphene a viable candidate material for large-scale applications. Graphene’s versatility for chemical functionalization, such as covalent bonding, makes this material suitable for developing rational strategies for obtaining the highly ordered nanoarchitectures with improved optical and electronic functionalities [35,36,37,38].

As a photoactive component, we employed a novel genetically engineered PSI complex from a volcanic microalga *Cyanidioschyzon merolae* (*C. merolae*). This extremophilic red alga is a model organism for understanding the evolution of cell division and the intracellular transport machinery [39,40] due to the simplicity of the cell structure and genome composition. It is also an evolutionary intermediate between the prokaryotic (cyanobacteria) and eukaryotic (higher plants and algae) phototrophs [41]. The PSI biophotocatalyst from *C. merolae* was shown to be highly robust across a wide range of pH, temperature and illumination conditions [41]. For the oriented and stable immobilization of the PSI biophotocatalyst, an His_6_-tag was genetically engineered at the *N*-terminus of the stromal PsaD subunit of PSI, allowing for the preferential binding of PSI with its reducing side towards the graphene monolayer. This approach yielded a robust and ordered bionanoarchitecture designed to generate an efficient DET pathway between the graphene, two types of metal redox centers in the organic SAM and photo-oxidized PSI biophotocatalyst.

## 2. Results and Discussion

### 2.1. Genetic Engineering of the C. merolae His_6_-Tagged PsaD-PSI Biophotocatalyst

The PSI biophotocatalyst was genetically modified at the *N*-terminus of the extrinsic PsaD subunit to introduce an His_6_-tag in the vicinity of the F_B_ cluster (terminal electron acceptor) for anchoring the PSI complex onto SLG. The overarching aim was to achieve the homogenous and dense surface coverage with PSI together with a minimized distance between the F_B_ cluster and the electrode surface and a maximized power output by the formation of a highly ordered, yet simple, supramolecular nanoarchitecture.

**Scheme 1:**
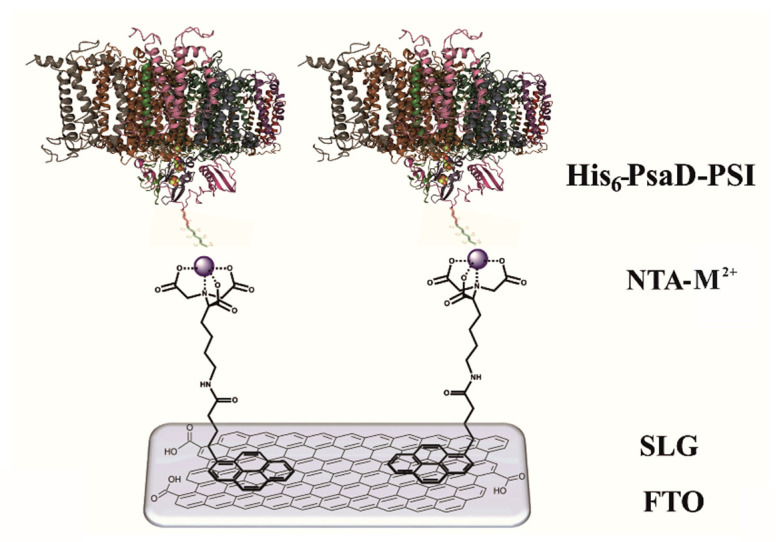
Diagrammatic representation of the full nanoarchitecture of the SLG/pyr-NTA-M^2+^/His_6_-PsaD-PSI nanoconstruct. M^2+^ redox center in the organic interface corresponds to Co^2+^ or Ni^2+^ cations used in this study.

The PSI immobilization occurred via the organic conductive interface based on the pyr-NTA SAM into which two distinct metal redox centers were incorporated (Ni or Co) as a rational strategy to improve DET (Scheme 1). A similar approach was used before for fine-tuning DET (in terms of kinetics and directionality) within graphene-based bioelectrodes incorporating cyt *c*_553_ and PSI electroactive components [38,42,43,44,45,46].

The innovative strategy for the generation of the recombinant His_6_-PsaD-PSI complex was based on importing the modified nuclear-encoded His_6_-PsaD protein into the chloroplast by the insertion of the chloroplast signaling peptide upstream of the 5′ region of the *psaD* sequence, then reconstituting the His_6_-PsaD-PSI complex in vivo, i.e., during the natural PSI turnover. This approach yielded the hybrid recombinant cell lines expressing both the native PSI and His_6_-PsaD-PSI complexes which could be separated during the subsequent purification procedure using the IMAC technology for capturing the His_6_-tagged proteins on the Ni-NTA matrix (see Materials and Methods).

**Scheme 2:**
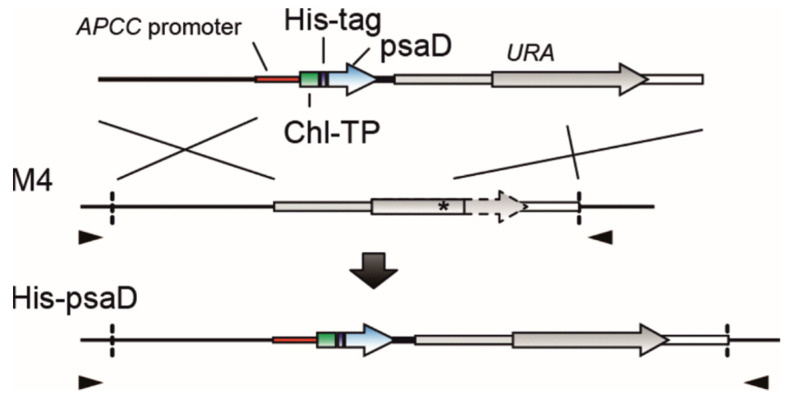
Graphic representation of the modified *psaD* (CMV144CT) gene inserted by homologous recombination in the upstream region of the URA locus. The first construct indicates introduced linear DNA, the second construct shows the genomic structure of the *C. merolae* uracil-auxotrophic mutant M4 and the third construct depicts the genomic structure of the *C. merolae* transformant expressing His_6_-PsaD protein. The asterisk shows the position of a frameshift mutation in the URA gene resulting in truncation of the *C*-terminal half of the URA protein in the M4 mutant. To express the *his-psaD* gene, the promoter and nucleotide sequence encoding the chloroplast-targeting peptide (Chl-TP) of APCC and *β*-tubulin terminator were connected to the upstream and downstream regions of the nucleotide sequence encoding the His_6_-PsaD protein. The arrows indicate the position of the primers used to confirm the integration of the construct into the chromosome.

**Figure 1:**
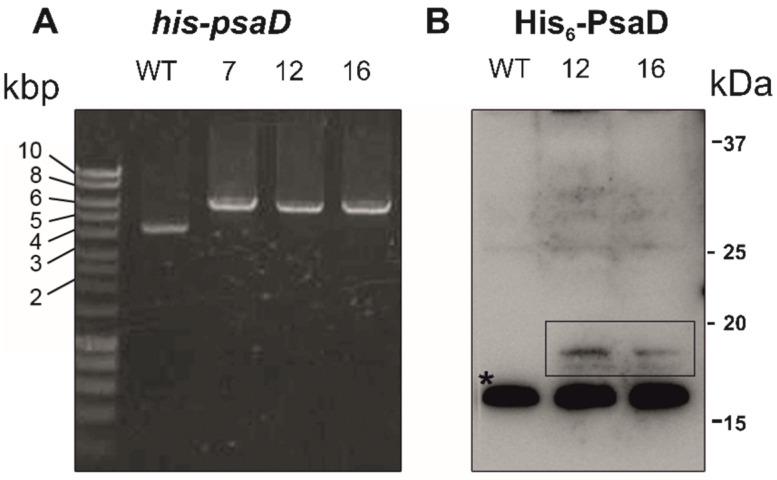
Characterization of the *C*. *merolae* recombinant cell lines expressing the nuclear encoded His_6_-PsaD protein. (**A**) PCR analysis of the three independent transformants (7, 12, 16). WT strain was used as a negative control. The position of the primers is shown in Scheme 1. (**B**) Western blot analysis of the total cell lysates of the two transformant lines (12 and 16) and the WT-negative control. The His_6_-tag was detected with anti-His_6_ monoclonal antibody, as described in Materials and Methods. The specific signals corresponding to the His_6_-PsaD bands are marked with a black box. Non-specific signals (marked with an asterisk) were used as a protein loading control.

The successful insertion of the recombinant *psaD* gene, carrying at the 5′ end the sequence for the chloroplast-targeting peptide and an *N*-terminal His_6_-tag (see Scheme 2), into the *C. merolae* nuclear genome (within an upstream region of the chromosomal URA locus) was confirmed by PCR (Figure 1).

Figure 1A shows the presence of a higher band of 5.7 kbp in the selected recombinant *C. merolae* cell lines (named 7, 12 and 16) compared to the lower PCR product of 4.3 kbp in the wild-type (WT) strain. These results confirm the presence of the recombinant *his-psaD* insert in the nuclear genome. Furthermore, the accumulation of the modified His_6_-PsaD protein inside the *C. merolae* cells was confirmed by Western blotting using the His_6_-tag-specific antibody. Figure 1B shows the presence of a specific protein band with an apparent size of 17 kDa, as expected for the His_6_-tagged PsaD subunit (see Table 1). This band was detected only in the recombinant cell lines (12 and 16) and was absent in the wild type (WT) cell lysate, confirming the specificity of immunodetection.

### 2.2. Biochemical Characterization of the His_6_-PsaD-PSI Complex

**Figure 2:**
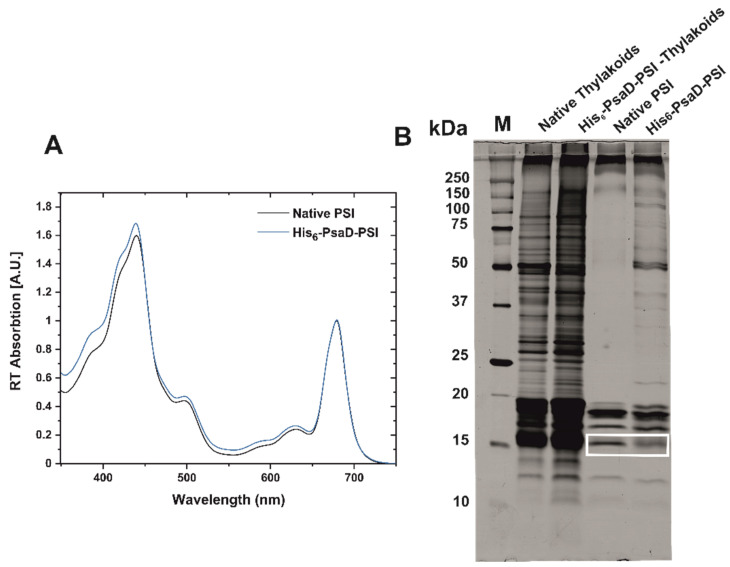
Biochemical characterization of the native PSI and His_6_-PsaD-PSI from *C. merolae*. (**A**) RT Absorption spectra of the native (black) and His_6_-PsaD-PSI (blue) complexes. (**B**) SDS-PAGE analysis of the native (thylakoids and WT PSI) and modified (thylakoids and His_6_-PsaD-PSI) proteins. The bands corresponding to the His_6_-tagged PsaD and native PsaD proteins are marked with a white box.

The purified His_6_-PsaD-PSI biophotocatalyst was biochemically and spectroscopically analyzed to confirm its homogeneity and intact photochemical activity following its solubilization from the thylakoid membranes. Figure 2A shows the absorption spectra of the recombinant and native PSI samples, with the Q_y_ peaks (originating from Chl*a*) detected for both samples at 679.5 nm, and the carotenoids peaks (originating mostly from *β*-carotene) detected at 439.5 nm for both types of preparations. The spectra were indistinguishable, confirming the identical spectroscopic properties and conformation of the recombinant PSI complex compared to its native counterpart [41,47].

The purity of the PSI samples was confirmed by the SDS-PAGE analysis. A typical PSI subunit pattern was observed, as shown by the comparison of the His_6_-PsaD-PSI and native PSI protein profiles (Figure 2B). These results confirmed the successful application of a one-step IMAC purification approach to obtain a pure and homogeneous His_6_-PsaD-PSI sample, similar to the His_6_-tagged PSI complexes purified to homogeneity from green algae and cyanobacteria [48,49]. The main core subunits of the PSI reaction center (PsaA/PsaB heterodimer) were not well defined due to the SDS-PAGE system applied for the preferential separation of low M_wt_ subunits, including the PsaD protein of 16–17 kDa. Interestingly, the His_6_-PsaD band appeared fuzzy compared to the well-defined native PsaD band, which could be due to the presence of the His_6_-tag (see bands inside the white box in Figure 2B).

To test the effect of the genetic introduction of an His_6_-tag into the PSI complex structure on the photochemical activity of this biophotocatalyst, the light-driven oxygen consumption activity of the His_6_-PsaD-PSI sample was compared to that of the purified native PSI complex. The photochemical activities were similar for both types of samples, with 1097 ± 95 µmol O_2_·mg Chl*a*^−1^·h^−1^ compared to 1137 ± 40 µmol O_2_·mg Chl*a*^−1^·h^−1^ for the His_6_-PsaD-PSI and native preparations, respectively (Figure S1). These values are also similar to the activity of the medium-light-grown native PSI complex from *C. merolae* reported by Haniewicz et al. (2018) [41] using a similar experimental setup for the PSI activity measurement. These results confirmed that the introduction of an His_6_-tag at the *N*-terminus of the PsaD subunit did not affect the activity of the recombinant PSI biophotocatalyst.

The mass spectrometry analysis confirmed the presence of all the PSI subunits in both types of samples (see Table S1 and S2, Supplementary Information), albeit the His_6_-PsaD-PSI preparation contained some contaminants over and above those detected for the native PSI complex sample, most likely due to the less stringent purification procedure. The overall coverage of the sequence for the PsaD subunit in both native and recombinant PSI samples was ~36%. However, in both cases, it was not possible to detect the last 5 amino acids of the *N*-terminal domain of the PsaD subunit, as this region was not amenable to proteolytic degradation (see Figure S2, Supplementary Information).

### 2.3. Confocal Microscopy

**Figure 3:**
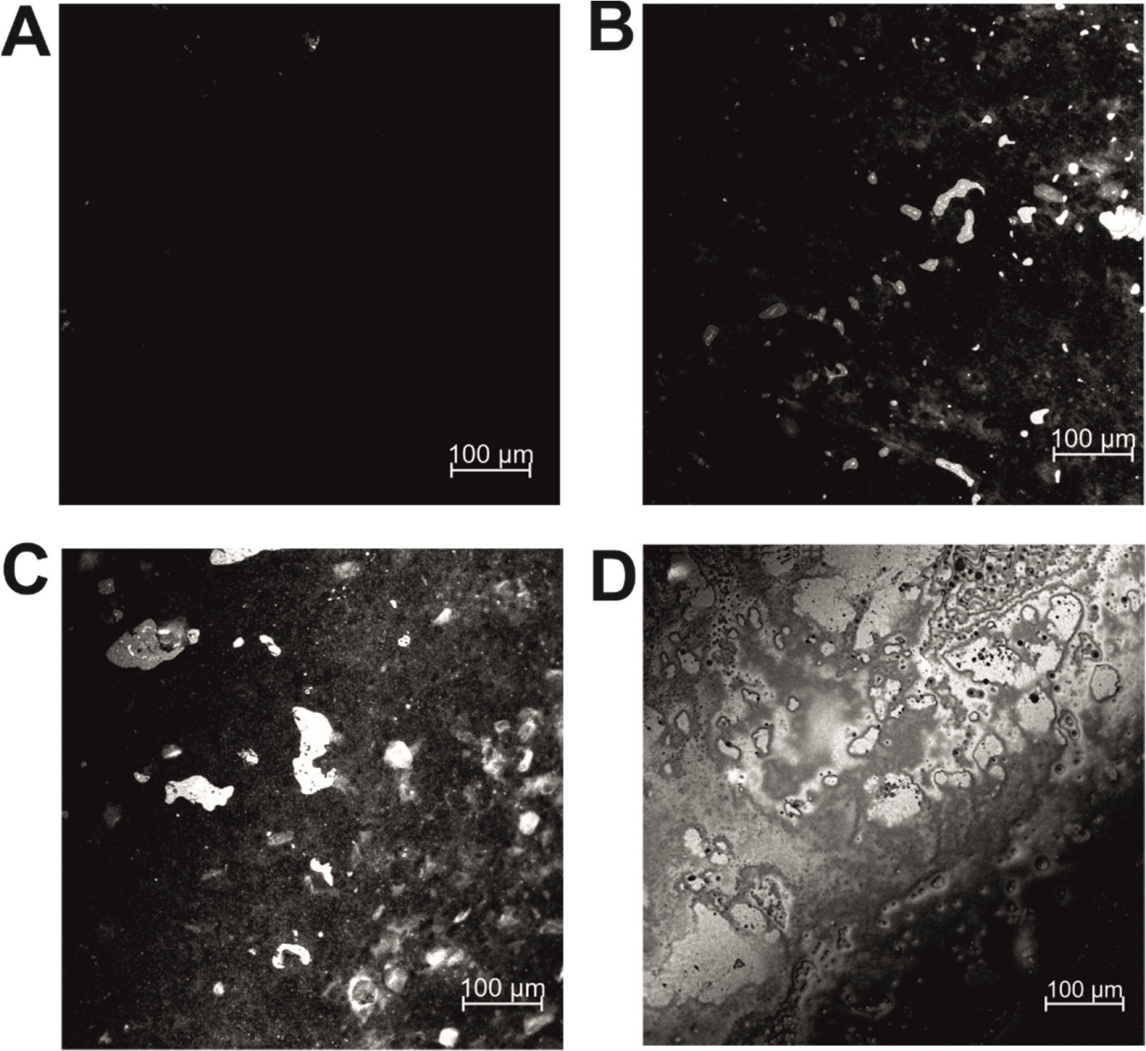
Confocal imaging of the PSI-biofunctionalized SLG/FTO electrodes. (**A**) FTO/SLG/pyr-NTA-Ni device used as a negative control. (**B**) FTO/SLG/pyr-NTA-Ni/PSI device. (**C**), FTO/SLG/pyr-NTA-Ni/cyt *c*_553_/PSI device, as analyzed in [37]. (**D**), FTO/SLG/pyr-NTA-Ni/His_6_-PsaD-PSI sample. The visualized surface area was ~0.41 mm^2^. Excitation was at 639 nm. Scale bar is 100 µm.

Three types of the biofunctionalized devices were prepared and analyzed via confocal microscopy to visualize the surface coverage of SLG with the recombinant versus native PSI biophotocatalyst (Figure 3). The control sample (Figure 3A) was the FTO/SLG/pyr-NTA-Ni electrode devoid of the biocomponent. Three distinct bioconjugation strategies were used: (i) functionalization via an electrostatic/hydrophobic interactions of native (WT) PSI with SLG (Figure 3B); (ii) molecular recognition between the natural electron donor to PSI, His_6_-tagged cyt *c*_553_ (captured on SLG via its *C*-terminal His_6_-tag on the pyr-NTA-Ni SAM) and native PSI based on an electrostatic and hydrophobic interactions between both proteins (Figure 3C); (iii) conjugation via coordination bonds between two His residues of the His_6_-tag of the PSI complex (within the PsaD subunit) with the pyr-NTA-M^2+^ SAM (M^2+^ being Ni, as shown in Figure 3D; or Co, data not shown). For confocal imaging of the PSI fluorescence, all the samples were excited at a 639 nm wavelength, which corresponds to the Chl*a* absorption wavelength of the Q_y_ band of the PSI complex [38]. The confocal imaging of the excited PSI complex immobilized on the SLG surface clearly demonstrated that the strategy based on capturing the His_6_-PsaD-PSI biophotocatalyst on the SLG via the pyr-NTA-M^2+^ SAM was the most effective one of all the approaches used. Importantly, the direct conjugation of the His_6_-PsaD-PSI biophotocatalyst with the SLG (Figure 3D) yielded a more homogenous functionalization of the electrode surface, compared to the other functionalization approaches used (compare Figure 3B,C). To gain a better insight into the SLG surface coverage with PSI, the fluorescence intensity distribution was obtained by generating a 2.5D view (pseudo 3D fluorescence intensity height maps, see Figure S3) from the 2D projections. These analyses confirmed that the SLG/pyr-NTA-M^2+/^His_6_-PsaD-PSI samples were characterized by the highest biophotocatalyst loading per surface area.

### 2.4. Analysis of the Bioelectrode Surface Morphology

**Figure 4:**
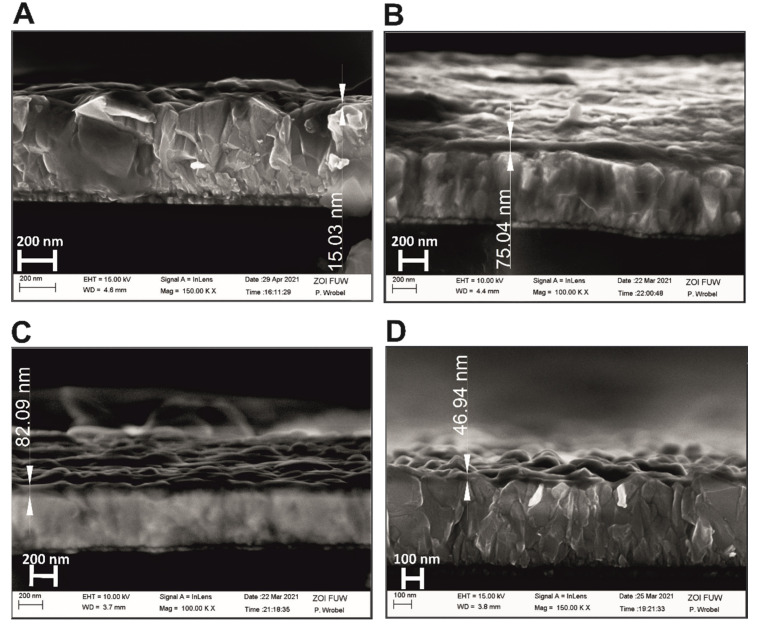
Cross-sectional imaging using scanning electron microscopy of glass/FTO/SLG electrodes functionalized with: (**A**) pyr-NTA-Ni SAM (d = 15 nm); (**B**) pyr-NTA-Ni/PSI (d = 97.5 nm); (**C**) pyr-NTA-Ni/cyt *c*_553_/PSI (d = 101 nm); and (**D**) pyr-NTA-Ni/His_6_-PsaD-PSI (d = 83 nm) nanoconstructs. d, cross-sectional thickness.

In order to visualize the electrode surface morphology, the bioelectrodes were analyzed via scanning electron microscopy (SEM) to estimate the thickness of the PSI biolayer developed using the above-mentioned bioconjugation strategies (Figure 4). The control sample was the FTO/SLG/pyr-NTA-Ni electrode (Figure 4A). All the biofunctionalized samples showed the formation of a multilayer of PSI, with an average thickness of 98 ± 53 nm for the physisorbed native PSI layer (Figure 4B), 101 ± 48 nm for the His_6_-cyt *c*/native PSI bioconjugate (Figure 4C) and 83 ± 74 nm for the His_6_-PsaD-PSI layer (Figure 4D). According to the SEM data, taking into account that the thickness of the PSI complex in the membrane plane was 10.6 nm [50], for all the biofunctionalized nanodevices the average number of the PSI monolayers in the multilayer was estimated as ~8–9 [38]. As expected, the SEM analysis confirmed a similar thickness for the PSI biolayer in all the types of samples analyzed in this study since a similar drop casting methodology was used for the biofunctionalization step. However, the best biophotocatalyst loading was observed for the direct conjugation approach, as shown by the highest fluorescence intensity per surface area for the His_6_-PsaD-PSI-based SLG sample (see Figure 3D).

### 2.5. Photoelectrochemical Characterization of the FTO/SLG/pyr-NTA-M^2+^/His_6_-PsaD-PSI Biophotoelectrodes

**Figure 5:**
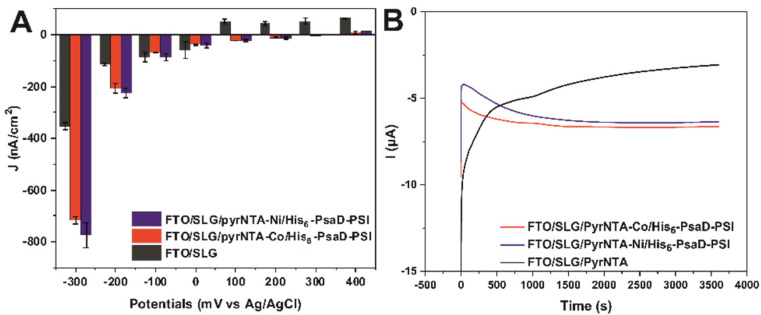
Photochronoamperometric analysis of the biohybrid His_6_-PsaD-PSI based nanoassemblies. (**A**) Photocurrent densities obtained from the freshly prepared FTO/SLG/pyr-NTA-Ni/His_6_-PsaD-PSI and FTO/SLG/pyr-NTA-Co/His_6_-PsaD-PSI samples under 30 s ‘light ON/OFF’ cycles vs. FTO/SLG control. These data were obtained for 2 independent samples (n = 2). (**B**) Photocurrent curves recorded for the representative FTO/SLG/pyr-NTA-Ni/His_6_-PsaD-PSI and FTO/SLG/pyr-NTA-Co/His_6_-PsaD-PSI samples vs. FTO/SLG/pyr-NTA control. Photocurrents were recorded at −0.3 V during 1 h continuous illumination (100 mW·cm^−2^).

The photocurrent production was examined for two distinct nanoarchitectures encompassing two metallic redox centers (Ni and Co) incorporated into the pyr-NTA SAM, in the presence or absence of oxygen. The functionalized FTO/SLG/pyr-NTA-M^2+^/His_6_-PsaD-PSI bioelectrodes, containing either Co or Ni cations in the organic interface, were characterized by photochronoamperometry to evaluate the photocurrent densities (J) (Figure 5A) as well as the photocurrent production stability under continuous standard illumination for 1 h (Figure 5B). The experiments were performed in an oxygenated phosphate buffer (pH 7), without an additional electron mediator, over the range of potentials from +0.4 V to −0.3 V (vs. Ag/AgCl). The different metal-based biohybrid devices showed a strong tendency to generate the cathodic photocurrent at an external potential range from +0.3 V to −0.3 V, while at +0.4 V a small anodic current was recorded with respective values of 10 nA·cm^−2^ and 15 nA·cm^−2^ for Co- and Ni-containing samples (Figure 5A). At a potential range from +0.3 V to −0.2 V, the photogenerated cathodic current increased slowly reaching values around 200 nA·cm^−2^ at −0.2 V for both types of devices. Interestingly, at −0.3 V, a significant enhancement of photocurrent production was observed, with a current output of 716 nA·cm^−2^ and 774 nA·cm^−2^ for the Co- and Ni-based samples, respectively (see Figure S4 for photochronoamperometric curves). Compared to the bare FTO/SLG sample, the novel biophotoelectrode nanoarchitecture encompassing the genetically modified His_6_-PsaD-PSI biophotocatalyst exhibited a two-fold improvement of the cathodic photocurrent output at −0.3 V. The average photocurrent densities generated in the PSI-based electrodes over and above those recorded for the FTO/SLG control were 363 nA·cm^−2^ and 421 nA·cm^−2^ for the Co- and Ni-containing nanoassemblies, respectively.

Overall, the systems described in this work generated 2–7-fold higher photocurrent densities compared to those obtained for the PSI-based devices constructed with various types of electrode material, including planar gold [51], 3D reduced graphene oxide [52] or SLG [38]. On the other hand, the biohybrid system based on PSI conjugated with nitrogen-doped carbon nanotubes in a wired or non-wired configuration generated the photocurrent density of 625 nA·cm^−2^ at +0.3 V vs. Ag/AgCl in the presence of an external redox mediator [53], which is lower than for the mediatorless SLG/pyr-NTA-M^2+^/His_6_-PsaD-PSI system reported here (see Table 2).

The use of the anchoring His_6_-tag moiety genetically inserted into the PSI structure significantly facilitated the formation of a robust biophotocatalyst layer without impairing the photocatalytic activity of the PSI complex. Nevertheless, it was important to investigate the stability of the novel bionanosystems constructed in this study by measuring the overall photocurrent output during 1 h of continuous standard illumination. Both nanosystems (based on Co or Ni) showed a similar stability of photocurrent production (see Figure 5B). A small photocurrent increase was observed in both systems during the first 30 min of irradiation, after which the current production reached a stable plateau. The overall current output was 6.35 µA (16.5 µA·cm^−2^) for the Ni- and 6.64 µA (17.3 µA·cm^−2^) for the Co-based PSI/SLG nanoassemblies. In contrast, the photocurrent obtained for the control sample (FTO/SLG/pyr-NTA) steadily decreased during 1 h of continuous illumination down to 3 µA (7.16 µA·cm^−2^), as shown in Figure 5B. These data clearly point towards the beneficial role of the PSI biolayer on improving the stability of photocurrent generation in SLG.

**Figure 6:**
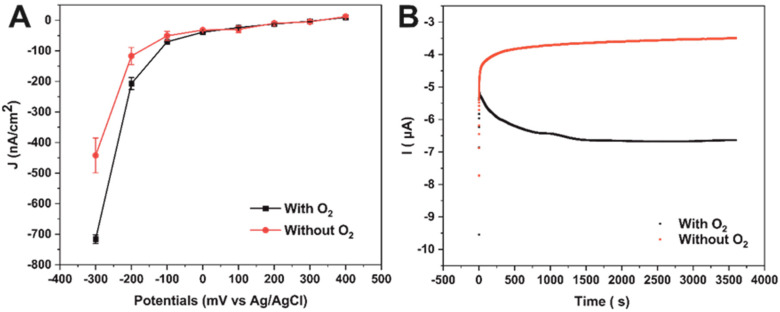
Photochronoamperometric analysis of the biohybrid FTO/SLG/pyr-NTA-Co^2+^/His_6_-PsaD-PSI nanoassemblies. (**A**) Photocurrent densities obtained from the freshly prepared samples in the presence (black) or absence (red) of oxygen under 30 s ‘light ON/OFF’ cycles. These data were obtained for 2 independent samples (n = 2). (**B**) Photochronoamperometric curves recorded for the representative FTO/SLG/pyr-NTA-Co/His_6_-PsaD-PSI sample at −0.3V during 1 h continuous illumination (100 mW·cm^−2^).

In order to gain a better insight into the mechanism of the photoinduced electron transfer, additional photochronoamperometric measurements were performed for the Co-based system in anoxic conditions (Figure 6). Oxygen is a well-known electron acceptor in photoelectric devices [23,54] and it can be reduced into the superoxide anion O^2−^ (E_m_ = −0.26 V vs. NHE) resulting in the enhancement of photocurrent production. The illumination of the PSI biophotocatalyst generated a charge-separate state followed by the formation of the reduced F_B_ cluster (E_m_ = −0.58 V vs. NHE) and the oxidized P700^+^ reaction center (E_m_ = +0.43 V vs. NHE). The reduced F_B_ cluster can, therefore, easily reduce the oxygen present in the electrolyte solution, while the oxidized P700^+^ will recover to the ground state upon the electron injection from the FTO/SLG electrode, leading to the generation of a cathodic current. Consequently, removing oxygen from the electrolyte solution resulted in a strong decrease in the total photocurrent output at −0.3 V with a value of 482 nA·cm^−2^ in anoxic vs. 716 nA·cm^−2^ in oxygenated conditions (Figure 6A). Interestingly, the absence of oxygen in the solution had a strong impact on the stability of the system and the overall current output during 1 h of continuous illumination (Figure 6B).

In anoxic conditions, the photocurrent started to diminish within 10 min of irradiation and continued to decrease resulting in the total current output two-fold lower compared to the photocurrent output in the presence of oxygen (3.45 µA vs. 6.64 µA).

## 3. Conclusions

This work reports a novel highly ordered photoactive nanoassembly based on the His_6_-PsaD-PSI biophotocatalyst, captured within the conductive organic SAM (pyr-NTA-M^2+^), on the graphene monolayer (SLG). This approach yielded the homogeneous and dense SLG coverage with the PSI biophotocatalyst leading to a significantly improved overall photocurrent output in conjunction with an enhanced stability of photocurrent generation compared to the abiotic FTO/SLG material. We used a rational approach to generate a highly ordered PSI nanoarchitecture, using an innovative genetic engineering of the PSI complex based on the cellular import of the nuclear-encoded His_6_-PsaD subunit into the chloroplast followed by the in vivo reconstitution of the PSI complex with the recombinant PsaD subunit. By generating the hybrid algal strain with both native PSI and His_6_-PsaD-PSI counterpart, an efficient approach was developed in this study for obtaining the homogenous and highly active His_6_-tagged PSI biophotocatalyst using the IMAC technology. The purified recombinant PSI biophotocatalyst proved suitable for the one-step functionalization of the SLG electrode surface modified with the His_6_-tag binding SAM. The His_6_-tag was genetically introduced into the structure of the stromal PsaD subunit, in the vicinity of the terminal electron acceptor, the F_B_ cluster, which greatly facilitated DET between the reducing side of PSI and SLG. Such an ordered bionanoarchitecture resulted in the generation of 2–7-fold higher overall photocurrents compared to those reported for the PSI-based devices encompassing graphenoids or gold likely due to: (i) the uniform orientation of the PSI complex on the electrode surface (with its reducing side towards SLG) promoting an efficient DET pathway between the graphene, metal center in the organic SAM and the F_B_ cluster; (ii) the minimization of the distance between the F_B_ cluster and SLG; (iii) the higher PSI photocatalyst loading per surface area compared to the other bioconjugation strategies based on PSI physisorption or the indirect binding of PSI via cyt c. The novel PSI/SLG nanosystem described in this work carries the high potential for future applications due to its robustness, ordered and simple architecture as well as the significantly enhanced simplicity of manufacturing.

## 4. Materials and Methods

### 4.1. Generation of the C. merolae Strain Containing His_6_-PsaD-PSI Biophotocatalyst

The genetic strategy to introduce the modified His_6_-tagged PsaD (CMV144CT, 417 bp) construct into URA locus in *Cyanidioschyzon merolae* M4 mutant was conducted as reported in Fujiwara et al. (2013) [55] with small modifications. Briefly, The M4 modified strain of *C. merolae* containing a point mutation in the encoding region of the chromosomal URA gene (1389 bp; CMK046C) was used which encodes orotidine 5′-phosphate decarboxylase. Due to the point mutation in the URA gene, the M4 mutants are uracil-auxotrophic and 5-fluoroorotic acid-resistant. Therefore, the wild-type URA introduced into M4 strain was used as a selective marker for genetic transformation. The stable expression of a transgene following linear DNA introduction into the nuclear genome was achieved by a polyethylene glycol method. For the introduction of an *N*-terminal His_6_-tag into the PsaD structure, the linear DNA, which consisted of the promoter and the sequence encoding the chloroplast-targeting peptide (Chl-TP) of APCC, His_6_-tagged full length *psaD* gene open reading frame (ORF) (see Table 1 for the amino acid sequences), *β*-tubulin terminator and URA5.3 selectable marker, were integrated into the nuclear genome. For the homologous recombination at the nuclear URA locus, the linear DNA described above was prepared so that it was sandwiched with the 5′- and 3′-chromosomal sequences flanking the URA locus, as described below (see Scheme 2).

First, the URA ORF flanked with the 2300 bp upstream and 471 bp downstream sequences was amplified by polymerase chain reaction (PCR) with the primer set #1/2 (see Supplementary Table S3) using *C. merolae* wild-type genomic DNA as a template, and cloned into a pGEMT-easy vector (Promega, Madison, MI, USA) using an In-Fusion HD Cloning Kit (Takara, Japan). The resultant plasmid pURA was amplified from the position at 898/897 bp upstream of the URA ORF by PCR with the primer set #3/4 (Supplementary Table S1) for the subsequent cloning. The 600 bp upstream flanking sequence of the *C. merolae* APCC ORF as the APCC promoter and a fragment corresponding to 1–180 nucleotides in the APCC ORF, encoding the chloroplast-targeting peptide (Chl-TP, 60 amino acids), were amplified by PCR with the primer set #5/6 (Supplementary Table S3). The codon-optimized sequence of the *his-psaD* gene was commercially synthesized (See Supplementary Table S3 for the nucleotide sequence). The downstream sequence of the *C. merolae* *β*-tubulin ORF (200 bp) as the terminator was amplified by PCR with the primer set #7/8 (Supplementary Table S3). The APCC promoter, Chl-TP, His_6_-psaD, and the *β*-tubulin terminator were assembled and cloned into a pURA plasmid between 898 bp and 897 bp upstream of the URA ORF using an In-Fusion kit. The resultant plasmid pAPCCp-Chl-TP-His_6_-psaD-URA was used as a template to amplify the linear DNA (for *C. merolae* transformation) using PCR with the primer set #9/10 (Supplementary Table S3). The final PCR product was introduced to the nuclear genome of *C. merolae* M4 mutant by homologous recombination (see Scheme 2). After the introduction of the PCR product, the algal transformants were selected on a uracil-free MA2 plate in a 5% CO_2_-containing incubator for 2 weeks until colonies appeared.

### 4.2. Culturing of C. merolae Cells and Isolation of Thylakoids Membranes

Cells of the wild-type and His_6_-PsaD-PSI *C. merolae* were cultivated in a standard Allen 2 medium, pH 2.5. The cells were grown at 42 °C, as described in Minoda et al. (2004) [56], with continuous white light illumination of 90 µE·m^−2^·s^−1^ (ML, Panasonic FL40SS-ENW/37 fluorescent light) and bubbling with 3–5% CO_2_, as described by Krupnik et al. (2013) [57] with small modifications. The 9 L cultures were grown to obtain the fresh biomass for PSI purification, as described in Haniewicz et al. (2018) [41] The thylakoids were isolated from the cell cultures grown to OD_680_ 0.9–1 according to the procedure reported in Haniewicz et al. (2018) [41]. Briefly, the cells were disrupted with glass beads (0.1 mm diameter) on ice for 13 cycles: 10 s ‘ON’ and 4 min ‘OFF’, in buffer A (10 mM CaCl_2_, 5 mM MgCl_2_, 25% (*w/v*) glycerol, 40 mM MES-NaOH, pH 6.1) supplemented with 5 mg of DNase I, 10 µL of RNase and 1 tablet of the protease inhibitor cocktail (Thermo Fisher Scientific, Waltham, MA, USA) per 50 mL of buffer A. The homogenate was filtered through Whatman^®^ filter paper, then ultra-centrifugated at 4 °C at 180,000× *g* for 25 min to collect thylakoid pellets. Thylakoids were washed 3 times with buffer A and collected by centrifugation as above. The thylakoid membranes were finally resuspended in buffer A, pH 6.1 and frozen in liquid nitrogen at a Chl*a* concentration of 2–5 mg·mL^−1^ prior to further use. Culturing of the wild-type *C. merolae *cells and the corresponding thylakoid isolation was performed as described in Haniewicz et al. (2018) [41].

### 4.3. Purification of His_6_-Tagged Proteins and Their Biochemical Characterization

The His_6_-PsaD-PSI complex was purified from the recombinant *C. merolae* thylakoids by the single-step metal affinity chromatography (IMAC) using 1 mL HisTrapTM HP column (Cytiva). Firstly, thylakoids were solubilized at a Chl*a* concentration of 1 mg·mL^−1^ with 1% (*w/v*) n-dodecyl-*β*-D-maltoside (DDM) in the dark on ice for 40 min. Prior to thylakoid loading, the HisTrapTM affinity column was equilibrated with 5 column volumes (CV) of the wash buffer (3 mM CaCl_2_, 0.03% (*w/v*) DDM, 25% (*w/v*) glycerol, 20 mM imidazole, HEPES-NaOH, pH 8). The solubilized and filtered thylakoid sample was loaded onto the HisTrapTM column at a 1 mL·min^−1^ flow rate, then washed with 10 CV of the wash buffer. The His_6_-PsaD-PSI complex bound to the column was eluted with a linear gradient of the elution buffer (3 mM CaCl_2_, 0.03% (*w/v*) DDM, 25% (*w/v*) glycerol, 1 M imidazole, 40 mM HEPES-NaOH, pH 8) at a flow rate of 1 mL·min^−1^. The eluate was collected in 2 mL fractions, with each fraction directly analyzed at room temperature (RT) via UV–VIS absorbance spectroscopy using a Shimazu UV–VIS 1800 spectrophotometer. The elution buffer was exchanged with the wash buffer devoid of imidazole by filtration. To this end, the elution buffer was diluted in 20 mL of wash buffer and exchanged using the Vivaspin20 devices (100 kDa MWCO, Sartorius) at 4000× *g* for 15 min. This step was repeated 3 more times. Purification of the native PSI complex was performed by 3-step anion exchange chromatography according to the procedure described in Haniewicz et al. (2018) [41]. The PSI samples were concentrated in the wash buffer devoid of imidazole (His_6_-PsaD-PSI) or devoid of NaCl (native PSI) to the final Chl*a* concentration of 2–5 mg·mL^−1^ using the Vivaspin 20 devices (100 kDa MWCO, Sartorius) at 2000 rpm, 4 °C (using a Eppendorf centrifuge 5430, rotor F-35-6-30), as described in Haniewicz et al. (2018) [41]. The concentrated samples were stored in small aliquots at −80 °C. The purity of the eluted fractions was confirmed by SDS-PAGE, as described in Rumak et al. (2012) [58]. Briefly, resolving polyacrylamide gradient gel was prepared using 14–20% (*w/v*) acrylamide and 0.2–0.4% (*w/v*) bis-acrylamide solution containing 12–17% (*w/v*) sucrose, 0.1% (*w/v*) SDS and 0.42 M Tris–HCl (pH 9.2). The stacking gel was prepared with 6% (*w/v*) acrylamide, 0.16% (*w/v*) bis-acrylamide, 0.1% (*w/v*) SDS and 54 mM Tris–HCl (pH 6.1). Thylakoid membranes and PSI samples were solubilized in a Laemmli sample buffer (Roti^®^Load 1, Roth GmbH) for 3 min at 95 °C. Samples containing 1 µg (thylakoids) or 2.5 µg (PSI preparations) of Chl*a* were loaded onto the gels and run overnight at a constant current of 8 mA using a Hoefer SE 400 electrophoresis system with a standard Tris-Glycine SDS buffer. Resolved gels were washed, then silver stained using a standard procedure [59]. The photochemical activity of PSI, measured by the oxygen consumption activity, was determined using an oxygen Clark-type electrode (Hansatech) following the methodology described elsewhere [41]. The native PSI and His_6_-PsaD-PSI purified samples (2 µg of Chl*a*) were analyzed at 30 °C in 40 mM HEPES-NaOH, pH 8 supplemented with 3 mM CaCl_2_, 0.03% (*w/v*) DDM and 25% (*w/v*) glycerol. Prior to the analysis 0.2 mM methyl viologen, 10 mM sodium azide and 0.2 mM dichlorophenolindophenol were added into the reaction mixture. After 2 min of dark incubation, 0.6 mM sodium ascorbate (sacrificial electron donor) was added to the reaction mixture, after which the solution was illuminated with 5000 µE·m^−2^·s^−1^ light intensity using a KL 2500 LCD lightbox (Schott). Three replicas were performed for each oxygen consumption measurement (n = 3).

Purification of the His_6_-cyt *c*_553_ protein (19AA peptide linker variant) and cyt redox activity measurements were performed as described in Kiliszek et al. (2018) [38].

### 4.4. LC-Mass Spectrometry Analysis

The Liquid Chromatography–Tandem Mass Spectrometry (LC-MS-MS) was performed on the native and the His_6_-PsaD-PSI samples, as described in Krupnik et al. (2013) [57]. Briefly, the PSI samples were precipitated in acetone and dissolved in 0.1% (*w/v*) RapiGestTM surfactant (Waters, Milford, MA, USA) in the presence of 50 mM ammonium bicarbonate. Following the reduction and alkylation of the Cys groups, the proteins were digested using sequencing grade trypsin (Sigma-Aldrich) overnight at 30 °C. Digested peptides were separated using a nanoAcquity Ultra Performance LC system connected to a SYNAPT G2 HDMS mass spectrometer (Waters). Peptides were loaded onto a Symmetry^®^ C18 (5 μm; 180 μm × 20 mm) trap column (Waters). The specifically captured peptides were then separated on a BEH 130 C18 analytical column (1.7 µm; 75 µm × 200 mm) (Waters). The elution was performed with a linear gradient of acetonitrile in 0.1% formic acid. Each sample was analyzed at least three times. Protein identification was performed using a ProteinLynx Global SERVER software (PLGS; version 2.2.5; Waters, Milford, MA, USA).

### 4.5. Preparation of Single Layer Graphene

Single layer graphene (SLG) was prepared as described previously [38]. The SLG production was performed using a copper foil via a chemical vapor deposition (CVD) method (see Supplementary Information for more details). The SLG was transferred onto Fluorine-doped Tin Oxide (FTO) substrate, which was precut to 1.5 × 1.5 cm squares. To verify the intactness and high quality of the obtained SLG, Raman scattering spectroscopy (Figure S5), Field emission-scanning electron microscopy (Figure S6), Polarized Light Microscopy (Figure S7), and Atomic Force Microscopy (Figure S8) analyses of the obtained samples were performed (see Supplementary Information).

### 4.6. FTO/SLG Electrode Biofunctionalization

The bioelectrode preparation was performed using a drop casting method as described in Kiliszek et al. (2018) [38]. Briefly, the FTO/SLG electrodes were sonicated for 1 min with acetone then air dried. The surface was overlaid with 30 µL of 2 mM pyrene-nitrilotriacetic acid (pyr-NTA) in *N*,*N*-dimethylformamide (DMF) solution in the dark at room temperature for 1 h. The excess of pyr-NTA was washed away with DMF [42]. The electrodes were air-dried overnight at RT, then incubated with 100 mM aqueous solution of nickel sulfate (NiSO4) or cobalt nitrate (Co(NO_3_)_2_) for 1 h at RT. The electrodes were rinsed 2–3 times with Milli-Q water and air-dried overnight at RT. Once fully dried, the electrodes were incubated with the purified His_6_-PsaD-PSI, native PSI, or cyt *c*_553_ (19AA linker variant [24]) followed by incubation with native PSI, as described in Kiliszek et al. [38] and Szalkowski et al. [43]. The biofunctionalization was performed overnight at 4 °C in the dark. The unbound proteins were removed by gently rinsing the electrodes with 5 mM phosphate buffer (pH 7), then air-dried at RT prior to use.

### 4.7. Confocal Microscopy Analysis

The biofunctionalized electrodes were analyzed using laser scanning confocal microscopy with a Zeiss LSM700 confocal microscope. The excitation wavelength was 639 nm, while the emission was recorded at 669 nm with a detection wavelength between 644 and 694 nm using a 50 nm range. Images were taken using the *Z*-stack mode with a range of 30.186 μm (6 slices in total) and acquired with a 10x objective (NA 0.3) using a pixel size of 0.63 µm and a pinhole size of 1 airy unit (AU). The sets of images taken at a various depth of focus were overlapped to generate a single picture with a greater depth of field (DOF) than any of the single source images. The obtained reconstituted image was used to build the 2.5-dimensional (2.5 D) fluorescence map using a ZEN 2.6 lite software.

### 4.8. Scanning Electron Microscopy Analysis

The cross-sectional imaging of the samples was performed with a Zeiss Sigma scanning electron microscope (SEM), high vacuum type with a field emission cathode (HV FE), equipped with in-lens secondary electron and backscattered electron detectors. The images were taken as a result of signal combination from the two detectors in a 50:50 percent ratio in order to maintain high visibility of the surface features and enhance the contrast of the material. To estimate the number of PSI layers on the different biofunctionalized electrodes, several SEM pictures were taken on different sections of each device. For the FTO/SLG/pyr-NTA-Ni control device, the SAM formed an ultrathin layer that was detectable with the maximum thickness of 15 nm. For the three types of biofunctionalized nanodevices, physisorbed native PSI, His_6_-tagged cyt *c*_553_/PSI and the His_6_-PsaD-PSI bionanoassemblies, the average thickness was calculated using the minimum and the maximum values recorded for each biolayer after subtracting the thickness obtained for the protein-free control.

### 4.9. Immunoblotting

Cells were harvested by centrifugation at 3000× *g* for 10 min at 4 °C. Cell pellets were solubilized with the sample buffer (2% SDS, 62 mM Tris-HCl pH 6.8, 100 mM DTT, 10% glycerol, and 0.01% Bromophenol Blue), then sonicated using a Cosmo Bio Bioruptor UCW-310 (settings: 310 W, 16 cycles of 30 s with a 30 s rest period in between). Total cellular proteins (3 µg per lane) were separated on 15% SDS-PAGE gels, then transferred onto polyvinylidene difluoride (PVDF) membranes (Immobilon, Millipore). The membrane blocking, antibody incubation, and signal detection were performed as described previously [60]. The anti-His_6_ monoclonal antibody (clone 2D8, #M136-3, MBL, Japan) was used at a dilution of 1:1000 to detect His-tagged proteins in the total cellular extracts.

### 4.10. Electrochemical Analyses

Photoelectrochemical analyses were performed using an AUTOLAB potentiostat/galvanostat (Metrohm Autolab B.V., PGSTAT128N) coupled to a KL 2500 LCD halogen white light source (Schott) with a standard light intensity (100 mW·cm^−2^; 1 sun). The biofunctionalized samples (1.5 cm × 1.5 cm) were used as a working electrode (WE) and were analyzed with a homemade 3-electrode electrochemical setup, with a working area of 0.4185 cm^2^. The electrolyte used for all the analyses was 5 mM phosphate buffer (pH 7). An Ag/AgCl in 3 M KCl solution was used as a reference electrode (RE), while a glassy carbon rod was used as counter-electrode (CE). Photocurrent measurements were performed in aerobic conditions after stabilizing the open circuit potential (OCP) of the WE, using a potential range from −300 mV to +400 mV (vs. Ag/AgCl) with 30 s ‘light ON/OFF’ cycles. The photocurrent measurements were performed on at least two independent samples (n = 2). Additional experiments were carried out on freshly prepared samples in the absence of oxygen, after bubbling the electrolyte for 20 min with argon.

## Figures and Tables

**Table 1:** Amino acid sequence of the His_6_-PsaD pre–pro-protein subjected to post-translation processing.

Domain	Amino Acid Sequence	Molecular Weight (kDa)
Chloroplast-targeting peptide (Chl-TP)	MFVQTSFFGTGVKASAKSAESQRCLAHSSWSVRMTGYDMNGSSAGNLGPRRIKSSGVAND	6.33
His_6_ epitope	MRGSHHHHHH	1.27
CMV144CT (*psaD* gene)	MLNLKMPSPSFLGSTGGWLRCAETEEKYAMTWSSDQQHIFEMPTGGAAVMNSGDNLLYLARKEQALALATQLRTQFKIQDYKIYRIFPSGEVQYLHPKDGVLPYQVNKGREQVGRVKSTIGKNVNPAQVKFTSKATYDR	15.68

**Table 2:** Comparison of photocurrent densities of PSI-based bio-hybrids devices.

Electrode Material	Photocurrent Density (nA·cm^−2^)	Presence of Redox Mediators	Applied Potential (V) (vs. Ag/AgCl)	Light Intensity (mW·cm^−2^)	Reference
Planar gold Electrode/TPDA-modified SAM/PSI	100	Yes	−0.1	80	[51]
Nanoporous gold leaf electrodes/TPDA-modified SAM/PSI	810	Yes	−0.1	80	[51]
FTO/SLG/pyr-NTA-Ni/His_6_-cyt *c*_553_/PSI	370	No	−0.3	100	[38]
3D reduced Graphene Oxide/PSI	480	No	−0.15	100	[52]
PS/Nitrogen-doped Carbon Nanotubes	625	Yes	+0.3	78	[53]
FTO/SLG/pyr-NTA-Ni/His_6_-PsaD-PSI	774	No	−0.3	100	This work

*The Average Photocurrent Density Values are Standardized for 30 s ‘Light ON/OFF’ Chronoamperometry Cycles.*

## Supplementary Materials

The following are available online at https://www.mdpi.com/article/10.3390/ijms22168396/s1, Figure S1: Photochemical activity of the *C. merolae* native and His_6_-tagged PSI samples; Table S1: LC-MS/MS analysis of the *C. merolae* His_6-_PsaD-PSI sample; Table S2: LC-MS/MS analysis of the native *C. merolae* PSI sample; Figure S2: Amino acid sequences of the *C. merolae* PsaD and His_6_-PsaD proteins superimposed with experimentally (MS/MS) identified peptides from the *C. merolae* PSI preparations; Figure S3: Confocal imaging of the PSI-biofunctionalized SLG/FTO electrodes; 2.5D fluorescence intensity maps; Figure S4: Photochronoamperometric curve obtained for FTO/SLG/pyr-NTA-M^2+/^His_6_-PsaD-PSI biohybrid device under 30 s. light/dark cycles; Table S3: Comparison of photocurrent densities of PSI-based bio-hybrids devices; Table S4: List of primers and a synthetic gene sequence used in the present study; Figure S5: Average Raman spectrum of a high-quality defect-free SLG on FTO substrate; Figure S6: Top-view SEM images of FTO substrate covered with SLG; Figure S7: Polarized light microscopy imaging of the FTO/SLG sample; Figure S8: AFM visualization of SLG on FTO surface.

## References

[1] Ort D.R., Good N.E. (1988). Textbooks ignore photosystem II-dependent ATP formation: Is the Z scheme to blame?. Trends Biochem. Sci..

[2] Barber J. (2004). Engine of Life and Big Bang of Evolution: A Personal Perspective. Photosynth. Res..

[3] Olah G.A. (2005). Beyond Oil and Gas: The Methanol Economy. Angew. Chem. Int. Ed..

[4] Solar FAQs.PDF. https://old-www.sandia.gov/~jytsao/Solar%20FAQs.pdf.

[5] Berry E.A., Guergova-Kuras M., Huang L.-S., Crofts A.R. (2000). Structure and Function of Cytochrome bc Complexes. Annu. Rev. Biochem..

[6] Hervás M., Navarro J.A., de la Rosa M. (2003). Electron Transfer between Membrane Complexes and Soluble Proteins in Photosynthesis. Acc. Chem. Res..

[7] Kargul J., Barber J. (2011). Structure and Function of Photosynthetic Reaction Centres. Molecular Solar Fuels.

[8] Kargul J., Kiliszek M. (2019). Artificial Photosynthesis. Bioelectrochemical Interface Engineering.

[9] Olmos J.D.J., Kargul J. (2015). A quest for the artificial leaf. Int. J. Biochem. Cell Biol..

[10] Brettel K., Leibl W. (2001). Electron transfer in photosystem I. Biochim. Biophys. Acta BBA Bioenerg..

[11] Teodor A., Bruce B.D. (2020). Putting Photosystem I to Work: Truly Green Energy. Trends Biotechnol..

[12] Nguyen K., Bruce B.D. (2014). Growing green electricity: Progress and strategies for use of Photosystem I for sustainable photovoltaic energy conversion. Biochim. Biophys. Acta BBA Bioenerg..

[13] Fang X., Kalathil S., Reisner E. (2020). Semi-biological approaches to solar-to-chemical conversion. Chem. Soc. Rev..

[14] Witt H.T. (1996). Primary reactions of oxygenic photosynthesis. Ber. Bunsenges. Phys. Chem..

[15] Nelson N., Yocum C.F. (2006). Structure and function of photosystems i and ii. Annu. Rev. Plant Biol..

[16] Kargul J., Olmos J.D.J., Krupnik T. (2012). Structure and function of photosystem I and its application in biomimetic solar-to-fuel systems. J. Plant Physiol..

[17] Muller M.G., Slavov C., Luthra R., Redding K.E., Holzwarth A.R. (2010). Independent initiation of primary electron transfer in the two branches of the photosystem I reaction center. Proc. Natl. Acad. Sci. USA.

[18] Lisdat F. (2017). Trends in the layer-by-layer assembly of redox proteins and enzymes in bioelectrochemistry. Curr. Opin. Electrochem..

[19] Ocakoglu K., Krupnik T., Bosch B.V.D., Harputlu E., Gullo M.P., Olmos J.J., Yildirimcan S., Gupta R.K., Yakuphanoglu F., Barbieri A. (2014). Photosystem I-based Biophotovoltaics on Nanostructured Hematite. Adv. Funct. Mater..

[20] Ocampo O.E.C., Gordiichuk P., Catarci S., Gautier D.A., Herrmann A., Chiechi R.C. (2015). Mechanism of Orientation-Dependent Asymmetric Charge Transport in Tunneling Junctions Comprising Photosystem I. J. Am. Chem. Soc..

[21] Mikayama T., Iida K., Suemori Y., Dewa T., Miyashita T., Nango M., Gardiner A.T., Cogdell R.J. (2009). The electronic behavior of a photosynthetic reaction center monitored by conductive atomic force microscopy. J. Nanosci. Nanotechnol..

[22] Shen Y.-X., Saboe P.O., Sines I.T., Erbakan M., Kumar M. (2014). Biomimetic membranes: A review. J. Membr. Sci..

[23] Badura A., Guschin D., Kothe T., Kopczak M.J., Schuhmann W., Rögner M. (2011). Photocurrent generation by photosystem 1 integrated in crosslinked redox hydrogels. Energy Environ. Sci..

[24] Olmos J.D.J., Becquet P., Gront D., Sar J., Dąbrowski A., Gawlik G., Teodorczyk M., Pawlak D., Kargul J. (2017). Biofunctionalisation of p-doped silicon with cytochrome c553minimises charge recombination and enhances photovoltaic performance of the all-solid-state photosystem I-based biophotoelectrode. RSC Adv..

[25] Mukherjee D., Vaughn M., Khomami B., Bruce B.D. (2011). Modulation of cyanobacterial photosystem I deposition properties on alkanethiolate Au substrate by various experimental conditions. Colloids Surf. B Biointerfaces.

[26] Szewczyk S., Giera W., D’Haene S., van Grondelle R., Gibasiewicz K. (2016). Comparison of excitation energy transfer in cyanobacterial photosystem I in solution and immobilized on conducting glass. Photosynth. Res..

[27] Miyachi M., Yamanoi Y., Yonezawa T., Nishihara H., Iwai M., Konno M., Iwai M., Inoue Y. (2009). Surface Immobilization of PSI Using Vitamin K1-Like Molecular Wires for Fabrication of a Bio-Photoelectrode. J. Nanosci. Nanotechnol..

[28] Frolov L., Rosenwaks Y., Carmeli C., Carmeli I. (2005). Fabrication of a Photoelectronic Device by Direct Chemical Binding of the Photosynthetic Reaction Center Protein to Metal Surfaces. Adv. Mater..

[29] Kaniber S., Frolov L., Simmel F.C., Holleitner A.W., Carmeli C., Carmeli I., Caldas M., Studart N. (2010). Covalently Binding the Photosystem I to Carbon Nanotubes.

[30] Zhu W., Salles R., Miyachi M., Yamanoi Y., Tomo T., Takahashi H., Nishihara H.H. (2020). Photoelectric Conversion System Composed of Gene-Recombined Photosystem I and Platinum Nanoparticle Nanosheet. Langmuir.

[31] Novoselov K.S., Geim A.K., Morozov S.V., Jiang D., Zhang Y., Dubonos S.V., Grigorieva I.V., Firsov A.A. (2004). Electric field effect in atomically thin carbon films. Science.

[32] What Is Graphene? Graphene Properties and Applications. Nanowerk. https://www.nanowerk.com/what_is_graphene.php.

[33] Geim A.K., Novoselov K. (2007). The rise of graphene. Nat. Mater..

[34] Nair R.R., Blake P., Grigorenko A.N., Novoselov K., Booth T., Stauber T., Peres N.M.R., Geim A.K. (2008). Fine Structure Constant Defines Visual Transparency of Graphene. Science.

[35] Gunther D., Leblanc G., Prasai D., Zhang J.R., Cliffel D.E., Bolotin K.I., Jennings G.K. (2013). Photosystem I on Graphene as a Highly Transparent, Photoactive Electrode. Langmuir.

[36] LeBlanc G., Winter K.M., Crosby W.B., Jennings G.K., Cliffel D.E. (2014). Integration of Photosystem I with Graphene Oxide for Photocurrent Enhancement. Adv. Energy Mater..

[37] Feifel S.C., Stieger K.R., Lokstein H., Lux H., Lisdat F. (2015). High photocurrent generation by photosystem I on artificial interfaces composed of π-system-modified graphene. J. Mater. Chem. A.

[38] Kiliszek M., Harputlu E., Szalkowski M., Kowalska D., Ünlü G., Haniewicz P., Abram M., Wiwatowski K., Niedziółka-Jönsson J., Mackowski S. (2018). Orientation of photosystem I on graphene through cytochrome c553 leads to improvement in photocurrent generation. J. Mater. Chem. A.

[39] Kuroiwa T. (1998). The primitive red algae Cyanidium caldarium and *Cyanidioschyzon merolae* as model system for investigating the dividing apparatus of mitochondria and plastids. BioEssays.

[40] Miyagishima S.-Y., Nishida K., Mori T., Matsuzaki M., Higashiyama T., Kuroiwa H., Kuroiwa T. (2003). A Plant-Specific Dynamin-Related Protein Forms a Ring at the Chloroplast Division Site. Plant Cell.

[41] Haniewicz P., Abram M., Nosek L., Kirkpatrick J., El-Mohsnawy E., Olmos J.D.J., Kouřil R., Kargul J.M. (2018). Molecular Mechanisms of Photoadaptation of Photosystem I Supercomplex from an Evolutionary Cyanobacterial/Algal Intermediate. Plant Physiol..

[42] Osella S., Kiliszek M., Harputlu E., Ünlü G., Ocakoglu K., Kargul J., Trzaskowski B. (2018). Controlling the charge transfer flow at the graphene/pyrene–nitrilotriacetic acid interface. J. Mater. Chem. C.

[43] Szalkowski M., Harputlu E., Kiliszek M., Ünlü G., Maćkowski S., Ocakoglu K., Kargul J., Kowalska D. (2020). Plasmonic enhancement of photocurrent generation in a photosystem I-based hybrid electrode. J. Mater. Chem. C.

[44] Jacquet M., Izzo M., Osella S., Kozdra S., Michałowski P.P., Gołowicz D., Kazimierczuk K., Gorzkowski M.T., Lewera A., Teodorczyk M. (2021). Development of a universal conductive platform for anchoring photo- and electroactive proteins using organometallic terpyridine molecular wires. Nanoscale.

[45] Izzo M., Osella S., Jacquet M., Kiliszek M., Harputlu E., Starkowska A., Łasica A., Unlu C.G., Uśpieński T., Niewiadomski P. (2021). Enhancement of direct electron transfer in graphene bioelectrodes containing novel cytochrome c variants with optimized heme orientation. Bioelectrochemistry.

[46] Jacquet M., Kiliszek M., Osella S., Izzo M., Sar J., Harputlu E., Unlu C.G., Trzaskowski B., Ocakoglu K., Kargul J. (2021). Molecular mechanism of direct electron transfer in the robust cytochrome-functionalised graphene nanosystem. RSC Adv..

[47] Abram M., Białek R., Szewczyk S., Karolczak J., Gibasiewicz K., Kargul J. (2020). Remodeling of excitation energy transfer in extremophilic red algal PSI-LHCI complex during light adaptation. Biochim. Biophys. Acta BBA Bioenerg..

[48] Kubota H., Sakurai I., Katayama K., Mizusawa N., Ohashi S., Kobayashi M., Zhang P., Aro E.-M., Wada H. (2010). Purification and characterization of photosystem I complex from Synechocystis sp. PCC 6803 by expressing histidine-tagged subunits. Biochim. Biophys. Acta (BBA) Bioenerg..

[49] Gulis G., Narasimhulu K.V., Fox L.N., Redding K.E. (2008). Purification of His6-tagged Photosystem I from Chlamydomonas reinhardtii. Photosynth. Res..

[50] Jordan P., Fromme P., Witt H.T., Klukas O., Saenger W., Krauß N. (2001). Three-dimensional structure of cyanobacterial photosystem I at 2.5 &Aring; resolution. Natture.

[51] Ciesielski P.N., Scott A.M., Faulkner C.J., Berron B.J., Cliffel D., Jennings G.K. (2008). Functionalized Nanoporous Gold Leaf Electrode Films for the Immobilization of Photosystem I. ACS Nano.

[52] Morlock S., Subramanian S.K., Zouni A., Lisdat F. (2021). Scalable Three-Dimensional Photobioelectrodes Made of Reduced Graphene Oxide Combined with Photosystem I. ACS Appl. Mater. Interfaces.

[53] Kim I., Jo N., Yang M.Y., Kim J., Jun H., Lee G.Y., Shin T., Kim S.O., Nam Y.S. (2019). Directed Nanoscale Self-Assembly of Natural Photosystems on Nitrogen-Doped Carbon Nanotubes for Solar-Energy Harvesting. ACS Appl. Bio Mater..

[54] Singh A., Mandal S., Chen S., Liu M., Gisriel C.J., Carey A.-M., Yan H., Seo D.-K., Lin S., Woodbury N.W. (2021). Interfacing Photosystem I Reaction Centers with a Porous Antimony-Doped Tin Oxide Electrode to Perform Light-Driven Redox Chemistry. ACS Appl. Electron. Mater..

[55] Fujiwara T., Ohnuma M., Yoshida M., Kuroiwa T., Hirano T. (2013). Gene Targeting in the Red Alga *Cyanidioschyzon merolae*: Single- and Multi-Copy Insertion Using Authentic and Chimeric Selection Markers. PLoS ONE.

[56] Minoda A., Sakagami R., Yagisawa F., Kuroiwa T., Tanaka K. (2004). Improvement of Culture Conditions and Evidence for Nuclear Transformation by Homologous Recombination in a Red Alga, *Cyanidioschyzon merolae* 10D. Plant Cell Physiol..

[57] Krupnik T., Kotabová E., van Bezouwen L.S., Mazur R., Garstka M., Nixon P., Barber J., Kana R., Boekema E.J., Kargul J. (2013). A Reaction Center-dependent Photoprotection Mechanism in a Highly Robust Photosystem II from an Extremophilic Red Alga, *Cyanidioschyzon merolae*. J. Biol. Chem..

[58] Rumak I., Mazur R., Gieczewska K., Kozioł-Lipińska J., Kierdaszuk B., Michalski W.P., Shiell B.J., Venema J.H., Vredenberg W.J., Mostowska A. (2012). Correlation between spatial (3D) structure of pea and bean thylakoid membranes and arrangement of chlorophyll-protein complexes. BMC Plant Biol..

[59] Shevchenko A., Wilm M., Vorm O., Mann M. (1996). Mass Spectrometric Sequencing of Proteins from Silver-Stained Polyacrylamide Gels. Anal. Chem..

[60] Fujiwara T., Kanesaki Y., Ehirooka S., Eera A., Sumiya N., Eyoshikawa H., Tanaka K., Miyagishima S.-Y. (2015). A nitrogen source-dependent inducible and repressible gene expression system in the red alga *Cyanidioschyzon merolae*. Front. Plant Sci..

