# Supplementary material for: Development of a Novel Nanoarchitecture of the Robust Photosystem I from a Volcanic Microalga Cyanidioschyzon merolae on Single Layer Graphene for Improved Photocurrent Generation
Source: Int J Mol Sci. 2021 Aug 5;22(16):8396. doi: 10.3390/ijms22168396 (PMC8395140; doi:10.3390/ijms22168396)
Supplement: Supplementary file 1 [file ijms-22-08396-s001.zip › ijms-1279621-supplementary.pdf]

Supplementary Information

Development of a novel nanoarchitecture of robust photosystem I from a volcanic microalga *Cyanidioschyzon merolae* on single layer graphene for enhanced photocurrent generation

Miriam Izzo[1], Margot Jacquet[1], Takayuki Fujiwara[2], Ersan Harputlu[3], Radosław Mazur[4], Piotr Wróbel[5], Tomasz Góral[6], C. Gokhan Unlu[7], Kasim Ocakoglu[3], Shin-ya Miyagishima[2], Joanna Kargul[1*]

[1]Solar Fuels Laboratory, Center of New Technologies, University of Warsaw, Banacha 2C, 02-097 Warsaw, Poland.

[2]Department of Gene Function and Phenomics, National Institute of Genetics, Yata 111, Mishima, Shizuoka, 411-8540, Japan.

[3]Department of Engineering Fundamental Sciences, Faculty of Engineering, Tarsus University, 33400, Tarsus, Turkey

[4]Department of Metabolic Regulation, Institute of Biochemistry, Faculty of Biology, University of Warsaw, Miecznikowa 1, 02-096 Warsaw, Poland.

[5]Faculty of Physics, University of Warsaw, Pasteura 7, 02-093, Warsaw, Poland.

[6]Cryomicroscopy and Electron Diffraction Core Facility, Center of New Technologies, University of Warsaw, 02-097 Warsaw, Poland

[7]Department of Biomedical Engineering, Pamukkale University, TR-20070 Denizli, Turkey

[*]corresponding author: j.kargul@cent.uw.edu.pl (J. K.)

Table of contents

Photochemical activity of purified PSI samples 2

Proteomic analysis of the native and His$_6$-PsaD-PSI samples 2

Additional confocal microscopy analysis 5

Photocurrent measurements 6

List of primers used for DNA constructs 6

Table S3. List of primers and a synthetic gene sequence used in the present study 6

Single layer graphene preparation and characterization 7

Photochemical activity of purified PSI samples

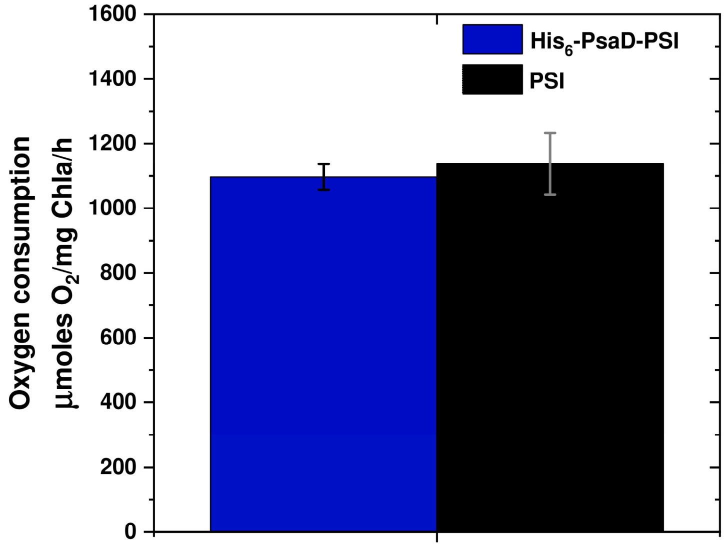

Figure S1. Photochemical activity of native and His6-tagged *C. merolae* PSI purified complexes. The values were obtained from three independent measurements and expressed as means ± SD.

Proteomic analysis of the native and His6-PsaD-PSI samples

Table S1. LC-MS/MS analysis of the *C. merolae* His6-PsaD- PSI. Proteins identified in the His6-PsaD-PSI complex preparation.

**MS-MS search results[a]**				
**Accession[b]**	**Protein name/description**	**PLGS score**	**Peptides found**	**Coverage [%]**
NP 848982.1	photosystem I subunit VII chloroplast	89614.8	7	70.4
NP 849051.1	photosystem I subunit II chloroplast	56379.8	16	42.4
NP 849104.1	photosystem I subunit III chloroplast	43701.2	11	44.9
XP 005537965.1	hypothetical protein CYME CMP346C	37623.1	15	52.4
NP 849064.1	allophycocyanin alpha subunit chloroplast	35229.4	12	72.7
NP 848987.1	phycocyanin beta chain chloroplast	33581.0	11	67.4
XP 005536140.1	mitochondrial F type ATPase F1 subunit beta precursor	26975.4	28	64.9
XP 005538084.1	similar to light harvesting protein	23590.0	19	45.3
XP 005539408.1	mitochondrial F type ATPase F1 subunit alpha precursor	22582.5	29	43.4
NP 848986.1	phycocyanin alpha chain chloroplast	22266.3	18	66.0
NP 849065.1	allophycocyanin beta subunit chloroplast	20084.3	12	62.1
XP 005537858.1	malate dehydrogenase mitochondrial precursor	19250.1	17	60.4
NP 849039.1	photosystem I subunit IV chloroplast	14593.4	7	56.4
NP 849045.1	photosystem I P700 chlorophyll a apoprotein A2 chloroplast	14385.7	31	21.2
XP 005537362.1	similar to chlorophyll a/b binding protein CP24	13370.2	3	17.1
XP 005535687.1	mitochondrial F1F0 ATP synthase subunit ATP5	10099.0	7	40.3
XP 005537833.1	phycocyanin-associated rod linker protein	9585.6	22	49.1
XP 005538967.1	eukaryotic translation initiation factor eIF 5A	9547.0	14	58.8
XP 005536581.1	mitochondrial F-type ATPase F0 subunit d	8397.6	6	45.4

XP 005537275.1	mitochondrial F-type ATPase F1 subunit delta precursor	6832.0	6	58.9
NP 849044.1	photosystem I P700 chlorophyll a apoprotein A1 chloroplast	6594.8	27	21.3
XP 005534978.1	hypothetical protein CYME CMJ266C	5827.5	7	36.5
XP 005537774.1	probable dehydrogenase	5277.2	23	47.0
XP 005538503.1	threonine 3-dehydrogenase	4633.7	10	35.0
NP 849135.1	photosystem I subunit XI chloroplast	4245.9	7	48.6
XP 005538061.1	hypothetical protein CYME CMQ114C	3997.7	10	43.9
XP 005539020.1	NADH dependent hydroxypyruvate reductase	3984.2	17	64.3
XP 005537353.1	mitochondrial F-type ATPase F1 subunit epsilon precursor	3601.6	2	37.3
XP 005538998.1	chloroplast 3-oxoacyl acyl carrier protein reductase precursor	3483.7	5	29.0
XP 005536151.1	heme oxygenase	3402.3	7	28.3
XP 005536698.1	mitochondrial F-type ATPase F1 subunit gamma precursor	2964.9	8	30.5
XP 005538360.1	hypothetical protein conserved	2906.2	18	37.3
XP 005538276.1	hypothetical protein CYME CMQ391C	2786.9	3	24.6
XP 005536091.1	succinyl CoA synthetase alpha chain	2542.2	7	25.8
XP 005536727.1	hypothetical protein CYME CML072C	2521.8	6	24.0
XP 005539234.1	succinate CoA ligase mitochondrial precursor	2452.3	11	38.2
XP 005538805.1	hypothetical protein CYME CMS143C	2341.3	2	18.3
XP 005535124.1	tryptophan tRNA ligase chloroplast or mitochondrial	2099.1	13	28.8
XP 005539433.1	similar to transmembrane calcium manganese transporter Ccc1p	1746.3	4	23.1
XP 005536306.1	single strand binding protein SSB	1320.6	8	40.3
NP 848983.1	glutamate synthase chloroplast	1305.7	25	21.9
XP 005538794.1	ATP dependent Clp protease ATP binding subunit ClpB	1287.2	19	25.6
XP 005538607.1	fusion protein of glyoxysomal fatty acid oxidation tetrafunctional protein and acetyl CoA acyltransferase	1241.3	20	24.5
XP 005536163.1	eukaryotic translation elongation factor 1 alpha	1240.0	7	24.0
XP 005537086.1	probable DNA binding protein	1227.1	8	29.7
XP 005536747.1	cytochrome *c* oxidase subunit Vb	1158.1	3	23.0
XP 005539325.1	serine threonine kinase KIN82	1137.9	8	27.7
XP 005536396.1	hypothetical protein CYME CMI242C	1085.7	2	16.1
NP 848974.1	phycobilisome rod core linker protein chloroplast	1064.7	8	34.6
NP 849063.1	phycobilisome linker protein chloroplast	861.9	15	25.1
XP 005537897.1	probable endothelin converting enzyme 1	844.5	11	20.1
XP 005537911.1	similar to cyclophilin B	843.7	3	15.8
XP 005536932.1	3-oxoacyl acyl carrier protein synthase II	826.6	3	12.6
XP 005535739.1	glutamate decarboxylase	776.5	5	14.8
XP 005538340.1	glucose-6-phosphate 1-dehydrogenase	763.8	7	13.7
XP 005537654.1	similar to nuclear receptor binding factor like protein	717.3	6	30.6
XP 005535431.1	small GTP binding protein of Rab family	677.0	5	26.8
XP 005534860.1	hypothetical protein CYME CMJ121C	626.6	1	11.5
XP 005536880.1	eukaryotic polypeptide chain release factor 3	614.6	4	11.1
XP 005534831.1	probable beta amylase	603.8	5	14.6
XP 005535849.1	mitochondrial intermembrane space complex subunit Tim10	569.4	1	23.9
XP 005534792.1	glyceraldehyde-3-phosphate dehydrogenase	521.0	8	34.7
XP 005538480.1	asparagine tRNA ligase chloroplast precursor	516.4	8	21.4
XP 005535297.1	similar to Transmembrane protein Tmp21 precursor	484.0	2	9.8

XP 005536254.1	similar to mitochondrial magnesium transporter Mrs2p	478.5	4	14.8
XP 005538846.1	glutamate tRNA ligase chloroplast or mitochondrial	440.6	6	12.3
XP 005535043.1	citrate synthase	427.1	8	21.5
XP 005538366.1	ferredoxin NADP reductase	418.4	4	18.1
XP 005538506.1	probable dehydrogenase	391.2	4	16.7
XP 005535272.1	glutathione reductase	387.4	4	12.9
XP 005537386.1	probable carotenoid cis trans isomerase CrtH	363.6	5	9.6
XP 005535558.1	photosystem II biogenesis protein Psb29	358.1	5	21.1
XP 005539125.1	hypothetical protein CYME CMT069C	325.1	5	11.7
XP 005535737.1	probable mannosyl oligosaccharide glucosidase	314.9	9	16.4
XP 005536533.1	acyl CoA oxidase ACX3	308.5	7	11.5
XP 005536821.1	serine protease	224.9	1	3.1

[a] *protein list generated by PLGS software using Cyanidioschyzon merolae protein RefSeq dataset (downloaded on April 1, 2021)*

[b] *RefSeq accession number*

Table S2. LC-MS/MS analysis of the native *C. merolae* PSI sample. Proteins identified in the native PSI complex preparation.

MS-MS search results[a]				
Accession[b]	Protein name/description	PLGS score	Peptides found	Coverage [%]
NP 848982.1	photosystem I subunit VII chloroplast	26482.0	12	71.6
NP 848986.1	phycocyanin alpha chain chloroplast	10568.1	19	66.0
NP 849051.1	photosystem I subunit II chloroplast	9588.0	19	36.0
NP 849104.1	photosystem I subunit III chloroplast	9094.6	11	44.9
NP 849135.1	photosystem I subunit XI chloroplast	7719.9	10	32.1
XP 005538084.1	similar to light harvesting protein	6539.9	15	34.1
NP 849045.1	photosystem I P700 chlorophyll a apoprotein A2 chloroplast	4336.3	34	21.3
XP 005537833.1	phycocyanin associated rod linker protein	4201.3	28	51.1
NP 849044.1	photosystem I P700 chlorophyll a apoprotein A1 chloroplast	2414.5	33	24.6
XP 005537362.1	similar to chlorophyll a/b binding protein CP24	2064.2	3	17.1
XP 005537522.1	outer mitochondrial membrane protein porin	1546.6	7	29.8
XP 005537773.1	hypothetical protein conserved	631.1	1	6.5
NP_848974.1	phycobilisome rod core linker protein chloroplast	425.3	5	25.6
XP_005536165.1	probable sulfate permease	362.3	5	7.3

[a] *protein list generated by PLGS software using Cyanidioschyzon merolae protein RefSeq dataset (downloaded on April 1, 2021)*

[b] *RefSeq accession number*

a

PsaD

**1**	MLNLK**MPSPS**	**FLGSTGGWLR**	CAETEEKYAM	TWSSDQQHIF	EMPTGGAAVM	NSGDNLLYLA
**51**	R**KEQALALAT**	**QLR**TQFKIQD	YKIYR**IFPSG**	**EVQYLHPKDG**	**VLPYQVNK**GR	EQVGRVKSTI
**111**	GKNVNPAQVK	FTSKATYDR				

b

His6-PsaD

**-10**						MRGSHHHHHH
**1**	MLNLK**MPSPS**	**FLGSTGGWLR**	CAETEEKYAM	TWSSDQQHIF	EMPTGGAAVM	NSGDNLLYLA
**51**	R**KEQALALAT**	**QLRTQFKIQD**	**YK**IYR**IFPSG**	**EVQYLHPKDG**	**VLPYQVNK**GR	EQVGRVKSTI
**111**	GKNVNPAQVK	FTSKATYDR				

Figure S2. Amino acid sequence of the *C. merolae* PsaD and His6-PsaD proteins superimposed with experimentally (MS/MS) identified peptides from *C. merolae* PSI preparations. **a,** Coverage map obtained from the native PSI sample. **b,** Coverage map obtained from His6-PsaD-PSI sample. Peptides from trypsin cleavage are denoted in bold.

Additional confocal microscopy analysis

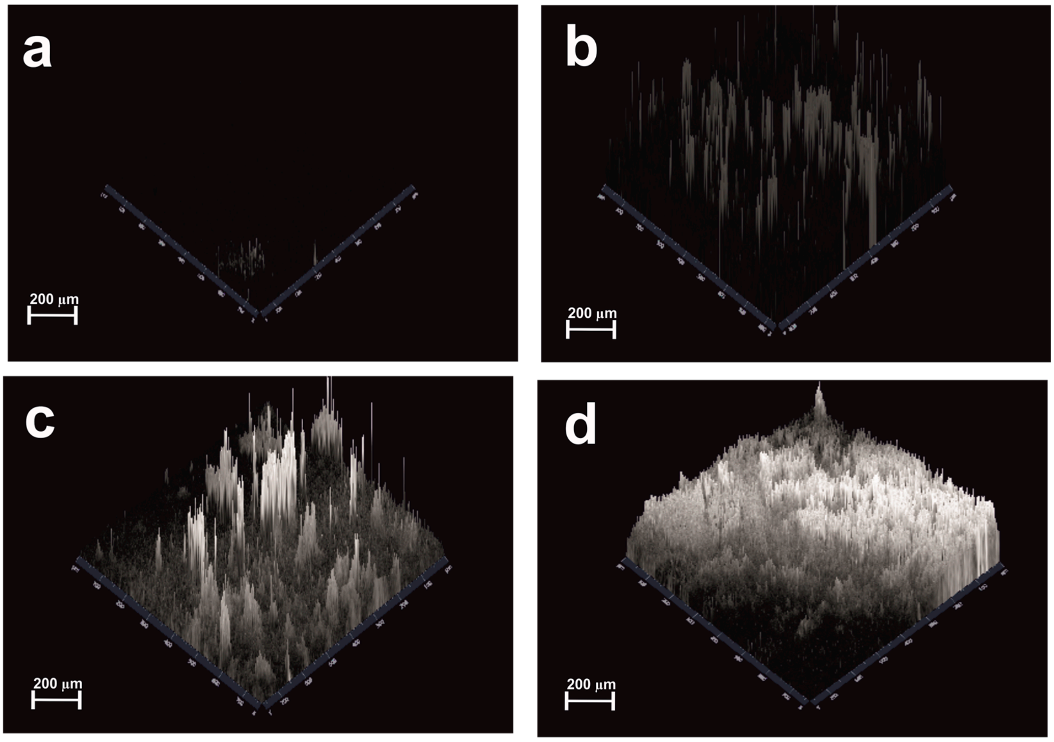

Figure S3. Confocal imaging of the PSI-biofunctionalized SLG/FTO electrodes, 2.5D fluorescence intensity maps. **a,** FTO/SLG/pyr-NTA-Ni device used as a negative control. **b,** FTO/SLG/pyr-NTA-Ni/PSI device. **c,** FTO/SLG/pyr-NTA-Ni/cyt c_{553}/PSI device. **d,** FTO/SLG/pyr-NTA-Ni/His6-PsaD-PSI device. The visualized area

is ~ 0.41 mm². Excitation is at 639 nm. Fluorescence distribution represented with a height map using an intensity range from 0 to 255, Scalebar is 200 µm.

Photocurrent measurements

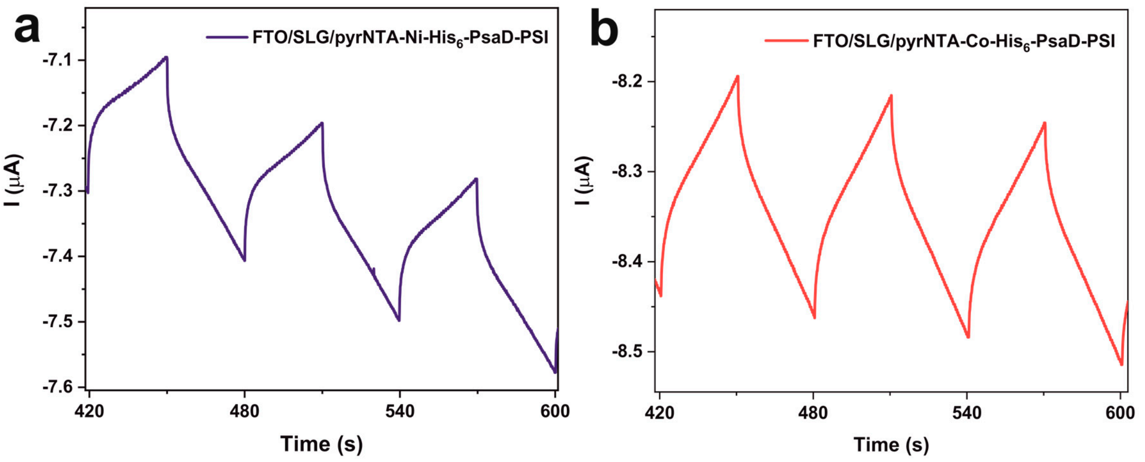

Figure S4. a, Photochronoamperometric curve obtained for FTO/SLG/pyr-NTA-Ni/His$_6$-PsaD-PSI biohybrid device under 30 s. light/dark cycles. **b**, Photochronoamperometric curve obtained for FTO/SLG/pyr-NTA-Co/His$_6$-PsaD-PSI biohybrid device under 30 s. light/dark cycles. Applied potential -0.3 V. Electrolyte: 5 mM phosphate buffer (pH 7.0). Light intensity: 100 mW cm^{-2} (1 sun).

List of primers used for DNA constructs

Table S3. List of primers and a synthetic gene sequence used in the present study.

No.	Name	Nucleotide sequence
1	URA(-2300)Fpgemt	ccgcgggaattcgatCTTCAAGAAAAGAGGATCTTTTGCCGTGATGCC
2	URA(+471)Rpgemt	gaattcactagtgatCCCTAGCAGCTGACTGTATCTCTATTCTTAGGAAT
3	URA(-898)R	AGTCATACAACAGTACTCAGATCGTTG
4	URA(-897)F	GAACTGAGGGGCGAACGCA
5	APCC(-600)Fura	tactgttgtatgactCGACGAGAACGTATAAGGAGTGC
6	APCC(180)Rhis	atgggagccacgcatATCATTCGCAACGCCAGATG
7	β-tubulinTer(+1)FpsaD	gccacgtacgaccggTAAACTAGCTATTTATCTGGTACATATCATTCAT
8	β-tubulinTer(+200)Rura	gaactgaggggcgaaACACTTTTTGCCTGCACAAG
9	pGEMTeasyF	CGTTGTAAAACGACGGCCAGT
10	pGEMTeasyR	ACAATTTCACACAGGAAACAGCTATGAC
	The lowercase letters indicate sequences for In-Fusion reaction	
9	His$_6$-PsaD	ATGCGTGGCTCCCATCACCATCACCACCACATGCTGAATC<br>TGAAGATGCCCTCCCCAAGCTTCTTGGGTTCGACTGGCGG<br>TTGGTTGCGCTGCGCGGAAACGGAAGAGAAATATGCGATG<br>ACGTGGTCGAGCGACCAGCAGCATATCTTCGAGATGCCGA<br>CGGGTGGGGCAGCCGTTATGAACTCAGGCGATAATCTCCTT<br>TATCTCGCTCGCAAAGAGCAGGCGCTGGCTCTGGCAACACA<br>ACTCCGCACACAGTTTAAGATCCAGGATTACAAGATCTATC<br>GTATTTTTCCATCGGGCGAAGTTCAATACCTCCACCCGAAGG

ACGGCGTGCTTCCCTACCAGGTGAACAAAGGGCGAGAGCAA
GTAGGACGGGTCAAAAGTACCATTGGAAAGAACGTGAACCC
GGCCCAAGTCAAGTTCACCTCGAAGGCCACGTACGACCGG

Single layer graphene preparation and characterization

The graphene layer was grown on the Cu foil, then transferred onto the fluorine-doped tin oxide (FTO) substrate as described below. Firstly, a polymethyl methacrylate (PMMA) solution coated onto the SLG/Cu surface by spin coating. Then, PMMA coated SLG/Cu substrate keep in the iron (III) nitrate solution for 1 day to etch Cu substrate. The PMMA/graphene layer was washed with deionized water and hydrochloric acid (HCl) solution to remove metal contaminants, then washed again in deionized water. The graphene-PMMA layer was transferred onto the FTO-coated glass surface. The PMMA/graphene/FTO substrate was left in 99.9% pure acetone at 40 °C for 30 min to remove the PMMA layer. The resultant SLG on the FTO surface was analyzed by Raman scattering spectroscopy using a WITec alpha300 Raman microscope to determine the number of graphene layers, the density of possible defects in the SLG structure, and the presence of undesirable impurities. Figure S1 presents a typical Raman spectrum of the SLG on the FTO substrate. The D, G, and 2D peaks were identified following excitation of SLG on FTO substrates at 488 nm and were determined at 1332.70, 1582.96 and 2658.70 cm^{-1} (see Fig. S1). The ratio of the D band to the G band, which is commonly used to assess the quality of a graphene layer, was 0.11, which indicated that very low amount of defects in the π-system and the high quality of SLG transferred onto the FTO substrate. Moreover, a sharp and symmetrical 2D band in conjunction with the high 2D/G peak ratio of 1.71 (Fig. S1) confirms the formation of SLG in our system.

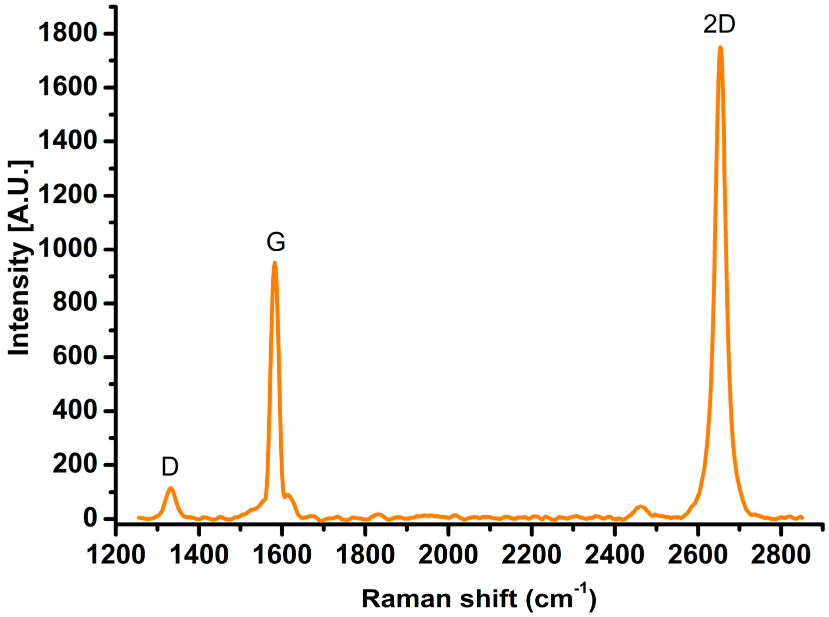

Figure S5. Average Raman spectrum of a high-quality defect-free SLG on FTO substrate. The D, G, and 2D bands represent the main Raman features of a high-quality graphene layer.

The Field emission-scanning electron microscopy (Zeiss/Supra 55 FE-SEM) images (Fig. S2) and light microscopy (Fig. S3) analyses confirmed that the SLG completely covers the surface of the FTO substrate. From the SEM images it can be concluded that the transparent graphene layer made the morphology of the FTO surface visible.

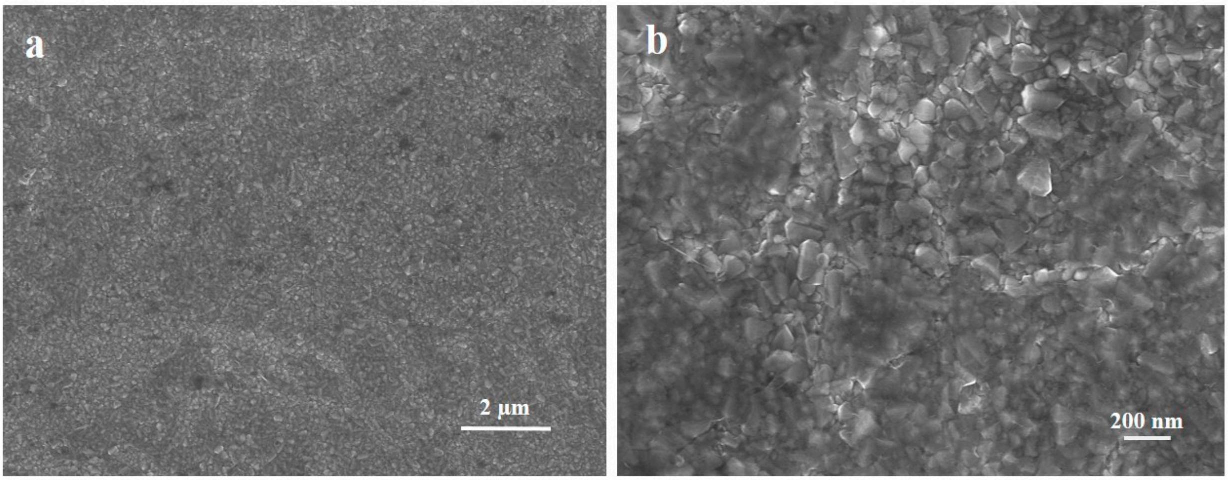

Figure S6. Top-view SEM images of FTO substrate covered with SLG. Panels (a) and (b) show FTO surface coated with SLG visualized at two different magnifications, as indicated.

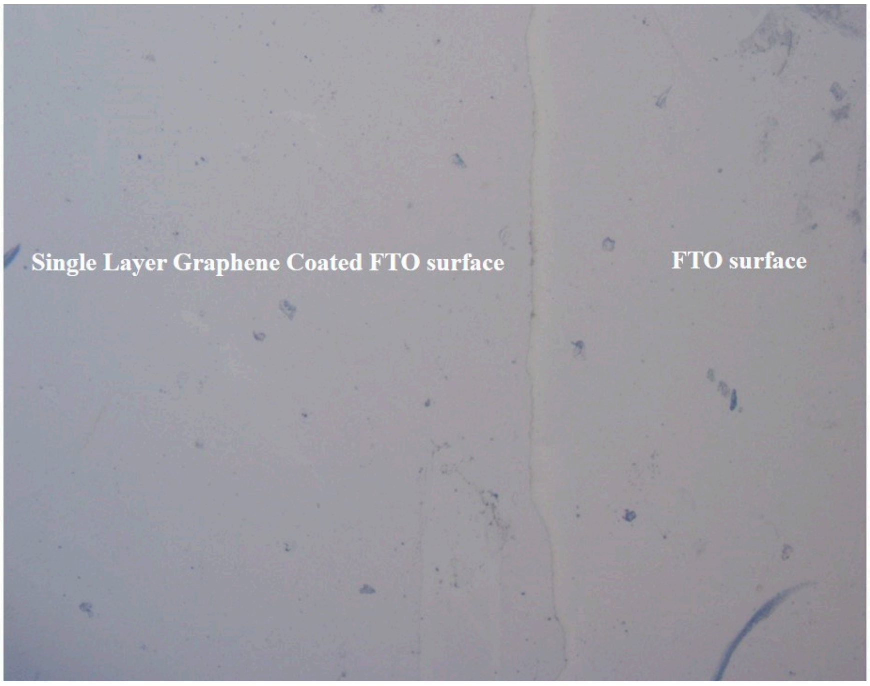

Figure S7. Polarized light microscopy imaging of the FTO/SLG sample. The polarized light micrograph shows areas fully covered with SLG and bare FTO.

Atomic Force Microscopy (AFM) measurements were carried out in a non-contact mode using a Park System XE-100 SPM instrument under ambient conditions. Furthermore, as seen in the AFM image of the SLG/FTO electrode (see Fig. S4), deposition of graphene substantially reduces the roughness of the electrode surface. The red scan line took from SLG/FTO surface and the average roughness was measured near 18 nm, on the other hand, the green scan line took from just the FTO surface and the average roughness was measured ca. 35 nm.

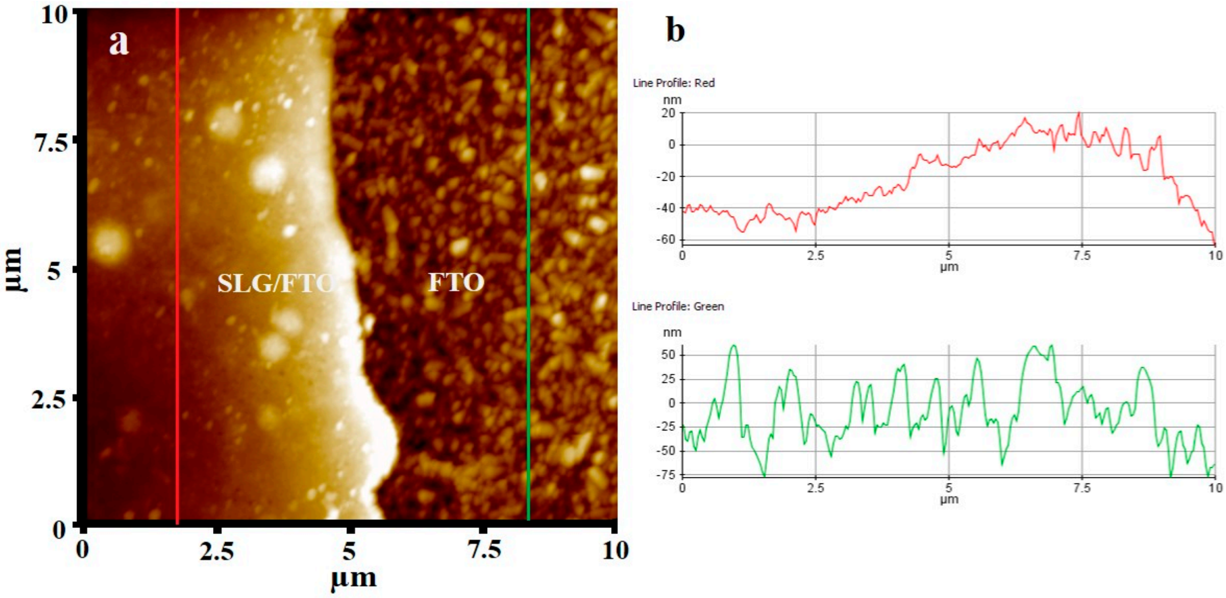

Figure S8. AFM visualization of SLG on FTO surface. Shown is a topographic image of SLG on FTO surface (a), as well as the AFM height profiles (b) of SLG/FTO surface (red line) and bare FTO surface (green line).
